# BACH1 promotes tissue necrosis and *Mycobacterium tuberculosis* susceptibility

**DOI:** 10.1038/s41564-023-01523-7

**Published:** 2023-12-08

**Authors:** Eduardo P. Amaral, Sivaranjani Namasivayam, Artur T. L. Queiroz, Eduardo Fukutani, Kerry L. Hilligan, Kate Aberman, Logan Fisher, Caio Cesar B. Bomfim, Keith Kauffman, Jay Buchanan, Leslie Santuo, Pedro Henrique Gazzinelli-Guimaraes, Diego L. Costa, Mariane Araujo Teixeira, Beatriz Barreto-Duarte, Clarissa Gurgel Rocha, Monique Freire Santana, Marcelo Cordeiro-Santos, Daniel L. Barber, Robert J. Wilkinson, Igor Kramnik, Kazuhiko Igarashi, Thomas Scriba, Katrin D. Mayer-Barber, Bruno B. Andrade, Alan Sher

**Affiliations:** 1 Immunobiology Section, Laboratory of Parasitic Diseases, NIAID, NIH, Bethesda, MD USA; 2 Laboratório de Inflamação e Biomarcadores, Instituto Gonçalo Moniz, Fundação Oswaldo Cruz (FIOCRUZ), Salvador, Bahia Brazil; 3 Immunology and Microbial Pathogenesis Program, Weill Cornell Medicine Graduate School of Medical Sciences, New York, NY USA; 4 T lymphocyte Biology Section, Laboratory of Parasitic Diseases, National Institutes of Allergy and Infectious Disease, National Institutes of Health, Bethesda, MD USA; 5 Helminth Immunology Section, Laboratory of Parasitic Diseases, National Institutes of Allergy and Infectious Disease, National Institutes of Health, Bethesda, MD USA; 6 Departmento de Bioquímica e Imunologia, Faculdade de Medicina de Ribeirão Preto, Universidade de São Paulo, Ribeirão Preto, Brazil; 7 Programa de Pós-Graduação em Imunologia Básica e Aplicada, Faculdade de Medicina de Ribeirão Preto, Universidade de São Paulo, Ribeirão Preto, Brazil; 8 Multinational Organization Network Sponsoring Translational and Epidemiological Research (MONSTER) Initiative, Salvador, Brazil; 9 Curso de Medicina, Universidade Salvador (UNIFACS), Laureate Universities, Salvador, Bahia Brazil; 10 Department of Pathology, School of Medicine of the Federal University of Bahia, Salvador, Bahia Brazil; 11 Center for Biotechnology and Cell Therapy, D’Or Institute for Research and Education (IDOR), Sao Rafael Hospital, Salvador, Bahia Brazil; 12 Departmento de Ensino e Pesquisa, Fundação Centro de Controle de Oncologia do Estado do Amazonas-FCECON, Manaus, Amazonas Brazil; 13 Fundação Medicina Tropical Doutor Heitor Vieira Dourado, Manaus, Amazonas Brazil; 14 Programa de Pós-Graduação em Medicina Tropical, Universidade do Estado do Amazonas, Manaus, Amazonas Brazil; 15 Faculdade de Medicina, Universidade Nilton Lins, Manaus, Amazonas Brazil; 16 Wellcome Centre for Infectious Disease Research in Africa, Institute of Infectious Disease and Molecular Medicine, University of Cape Town, Cape Town, South Africa; 17 The Francis Crick Institute, London, UK; 18 Department of Infectious Disease, Imperial College London, London, UK; 19 Boston University School of Medicine, Boston, MA USA; 20 Department of Biochemistry, Tohoku University Graduate School of Medicine, Sendai, Japan; 21 South African Tuberculosis Vaccine Initiative, Institute of Infectious Disease and Molecular Medicine and Division of Immunology, Department of Pathology, University of Cape Town, Observatory, South Africa; 22 Inflammation and Innate Immunity Unit, Laboratory of Clinical Immunology and Microbiology, NIAID, NIH, Bethesda, MD USA; 23 Curso de Medicina, Escola Bahiana de Medicina e Saúde Pública, Salvador, Bahia Brazil; 24 Faculdade de Medicina, Universidade Federal da Bahia, Salvador, Bahia Brazil; 25 Curso de Medicina, Universidade Faculdade de Tecnologia e Ciências (UniFTC), Salvador, Bahia Brazil; 26 Division of Infectious Diseases, Department of Medicine, Vanderbilt University School of Medicine, Nashville, TN USA

**Keywords:** Tuberculosis, Cell death and immune response, Cell death

## Abstract

Oxidative stress triggers ferroptosis, a form of cellular necrosis characterized by iron-dependent lipid peroxidation, and has been implicated in *Mycobacterium tuberculosis* (Mtb) pathogenesis. We investigated whether Bach1, a transcription factor that represses multiple antioxidant genes, regulates host resistance to Mtb. We found that BACH1 expression is associated clinically with active pulmonary tuberculosis. Bach1 deletion in Mtb-infected mice increased glutathione levels and Gpx4 expression that inhibit lipid peroxidation. *Bach1*^−/−^ macrophages exhibited increased resistance to Mtb-induced cell death, while Mtb-infected Bach1-deficient mice displayed reduced bacterial loads, pulmonary necrosis and lipid peroxidation concurrent with increased survival. Single-cell RNA-seq analysis of lungs from Mtb-infected *Bach1*^−/−^ mice revealed an enrichment of genes associated with ferroptosis suppression. Bach1 depletion in Mtb-infected B6.Sst1^S^ mice that display human-like necrotic lung pathology also markedly reduced necrosis and increased host resistance. These findings identify Bach1 as a key regulator of cellular and tissue necrosis and host resistance in Mtb infection.

## Abstract (web summary)

The host transcription factor, Bach1, promotes *Mycobacterium tuberculosis* pathogenesis by inhibiting protective glutathione metabolism and antioxidant responses that prevent ferroptosis.

## Main

Tuberculosis (TB) remains a major contributor to human mortality worldwide and in the wake of the coronavirus disease 2019 pandemic is once again the leading cause by a single pathogen^1^. *Mycobacterium tuberculosis* (Mtb), the causative agent of TB, most frequently targets the lungs initially infecting alveolar macrophages (AM)^2^. Disease progression is directly associated with subsequent pathogen dissemination mainly through necrotic death of infected myeloid cells^2–4^. In addition, cellular necrosis also plays an essential role in TB pathogenesis by promoting tissue inflammation. Thus, the elucidation of the pathways responsible for triggering cellular necrosis is important in both understanding TB disease and for the identification of candidate targets for host-directed therapy.

Previous studies have implicated a number of cell death mechanisms in the necrosis of Mtb-infected myeloid cells, including pyroptosis, necroptosis, NETosis as well as accidental cell death^5–8^, which may act concomitantly or redundantly to drive disease. In experimental murine models, cellular necrosis has also been linked to both type-I IFN production^9,10^ and lipid peroxidation driven by oxidative stress^11–15^. In turn, lipid peroxidation-mediated necrosis has been shown to be triggered by an iron-dependent process referred to as ferroptosis^16^ in which reactive oxygen species (ROS) attack host polyunsaturated fatty acid-enriched membranes, generating toxic forms of lipid peroxides that damage biological membranes. A major role for this pathway in TB is supported by the association of exacerbated oxidative stress response with worse disease outcomes in pulmonary TB patients (PTB) as well as in mice infected with Mtb^11,13,14^. A key molecule regulating ferroptosis is glutathione peroxidase-4 (Gpx4). This enzyme, which uses glutathione (GSH) as a co-factor, suppresses lipid peroxidation by reducing lipid peroxides to a non-toxic form, thus protecting cellular membranes from damage. Thus, changes in Gpx4 expression or activity can directly impact ferroptotic outcome. In this regard, persons with TB displaying more severe disease have been shown to exhibit reduced *GPX4* mRNA expression in circulating monocytes from peripheral blood mononuclear cells (PBMC) and lowered GSH levels in plasma^13,17,18^. Moreover, genetic depletion of Gpx4 in mice is associated with enhanced pulmonary bacterial burden as well as tissue necrosis and in vitro, promotes ferroptotic death of Mtb-infected macrophages^13^.

To avoid cellular damage due to exacerbated ROS production and maintain oxidative homoeostasis, host cells increase the transcription of antioxidant genes by promoting the activation of the nuclear factor erythroid 2–related factor 2 (Nrf2), a master transcription factor regulating expression of these genes^19^. In response to oxidative stress, Nrf2 dissociates from its cytosolic repressor Keap1 (Kelch-like erythroid cell-derived protein with CNC homology-associated protein 1), allowing its translocation to the nucleus where it induces genes related to GSH and iron metabolism by binding to antioxidant response elements (ARE) within their promoters^20^. In the case of murine Mtb infection, Nrf2 depletion has been shown to promote bacterial growth control within the first 10 days following aerosol exposure in vivo and has been identified as a key transcriptional network hub in AM in early infection^3^, while in humans, functional polymorphisms in the Nrf2 gene (*NFE2L2*) and lowered NRF2 protein levels are associated with increased susceptibility to active pulmonary TB disease^21^. Moreover, the ablation of genes induced by Nrf2, including *Gpx4*, *Fth1* and *Hmox1*, has been shown to increase murine susceptibility to Mtb infection^13,22,23^, suggesting an important general role for Nrf2-regulated antioxidant genes in host defence against this pathogen.

Interestingly, Nrf2 function is known to be suppressed by another transcription factor called BTB domain and CNC homologue 1 (Bach1). Bach1, a haem-binding protein, is widely expressed in mammalian tissues and in addition to regulating oxidative stress, it regulates multiple cellular processes such as GSH and iron metabolism, cell cycle and differentiation, mitochondrial metabolism, adipogenesis as well as immune responses^24^. Bach1 is considered as a pro-oxidant host factor because of its augmentation of ROS generation and its depletion is associated with protection from a number of different diseases/disorders, including atherosclerosis^25^, pulmonary fibrosis^26^, sepsis^27^, cardiomyopathy^28^, cancer^29^, spinal cord injury^30^, ischaemia/reperfusion injury^31^ and Parkinson’s disease^32^. Importantly, Bach1 has been recently reported to determine cell fate by acting as a major transcriptional regulator of ferroptosis through its effects on GSH and labile iron metabolism^28^.

In this study, we examine the role of Bach1 in regulating Mtb infection and pathogenesis. We demonstrate that Bach1 expression is elevated in TB patients as well as in lung granulomas from three different experimental animal models of Mtb infection. Furthermore, we show that Bach1 genetic ablation profoundly enhances host resistance to Mtb infection in mice as evidenced by reduced bacterial burden, pulmonary tissue lipid peroxidation and necrosis. Together, these findings provide important evidence supporting and further delineating the role of lipid peroxidation-mediated cell death and tissue necrosis in Mtb infection while implicating Bach1 as a potential target for host-directed therapy in tuberculosis.

## Results

### BACH1 is associated with pulmonary disease in TB patients

**Fig. 1:**
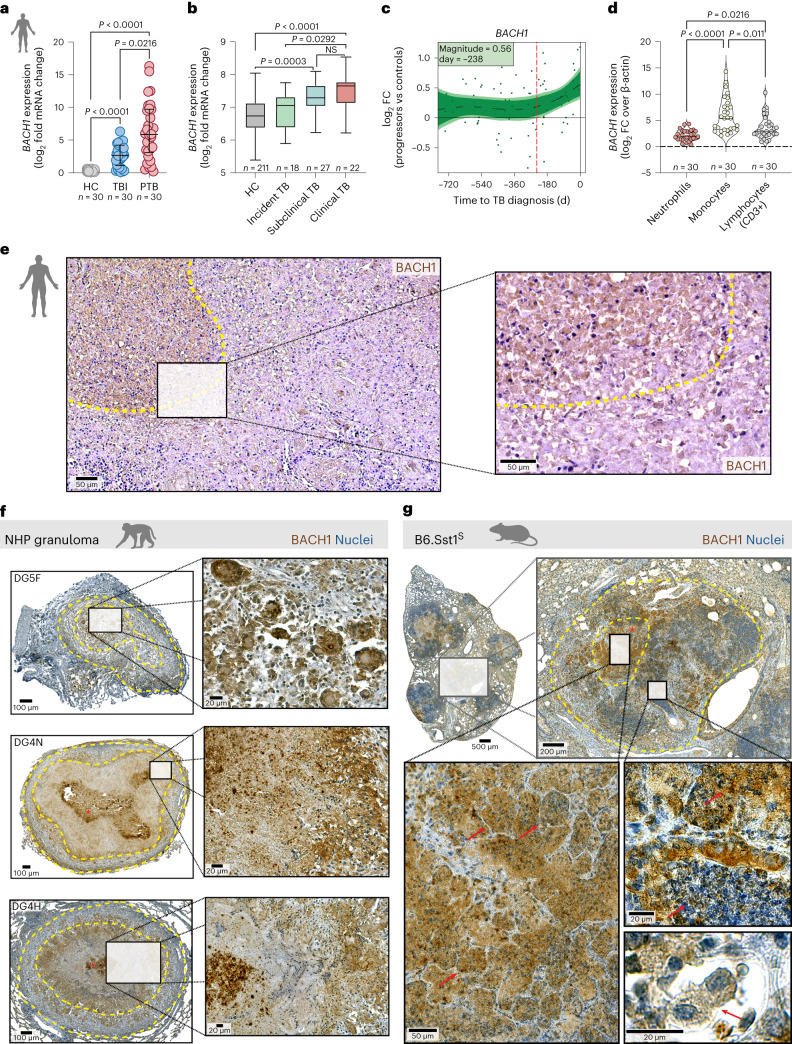
BACH1 expression is elevated in patients displaying active disease and is associated with pulmonary necrosis in animal models of TB. **a**,**d**, Plasma samples collected from PTB (*n* = 30), TBI (*n* = 30) and HC (*n* = 30) patients from a Brazilian cohort. **a**, *BACH1* mRNA expression in CD14^+^ monocytes (HC vs TBI, *P* < 0.0001; HC vs PTB, *P* < 0.0001; TBI vs PTB, *P* = 0.0216). **b**, *BACH1* mRNA expression in total PBMC isolated from patients from a South African cohort^34^. **c**, log_2_ fold change (FC) of *BACH1* mRNA expression in peripheral blood over time between bin-matched progressors (*n* = 44) and controls (*n* = 106) and modelled as nonlinear splines (black dashed line). Progressors are represented by light shading denoting 99% confidence interval (CI) and dark shading denoting 95% CI temporal trends. Dashed vertical line indicates the deviation time (day −238) calculated as the timepoint at which the 99% CI deviates from a log_2_ FC of 0. **d**, *BACH1* mRNA expression in sorted neutrophils, monocytes and lymphocytes from PTB patients (*n* = 29) (neutrophils vs monocytes, *P* < 0.0001; neutrophils vs lymphocytes, *P* = 0.0216; monocytes vs lymphocytes, *P* = 0.011). **e**, BACH1 staining in a post-mortem PTB patient lung section. Yellow dashed lines delineate area surrounding necrotic zone within the granuloma. Data represent median values and interquartile ranges. Statistical significance was determined using a two-sided Kruskal–Wallis test with post-hoc Dunn’s test for multiple comparisons. Boxplots shown in **b** represent median (centre line), upper and lower quartiles (box limits) and the interquartile range (whiskers). Data shown in **a**–**d** are presented as mean ± s.e.m. and each symbol denotes an individual patient. NS, not significant. **f**, BACH1 staining in lung sections from 3 rhesus macaques. The top left panel shows a cellular granuloma (delineated by yellow dashed line); middle and bottom left panels show necrotic granulomas (delineated by yellow dashed line) with the necrotic core outlined by yellow dashed line and highlighted by an asterisk. **g**, Representative lung sections from Mtb-infected B6.Sst1^S^ mice stained for Bach1. Dashed lines delineate the granuloma and asterisk indicates necrotic areas within the lesion. Red arrow points out cells resembling alveolar macrophages. Images shown are representative of those observed in 5 individual animals from 2 experiments. Source data

**Extended Data Fig. 1:**
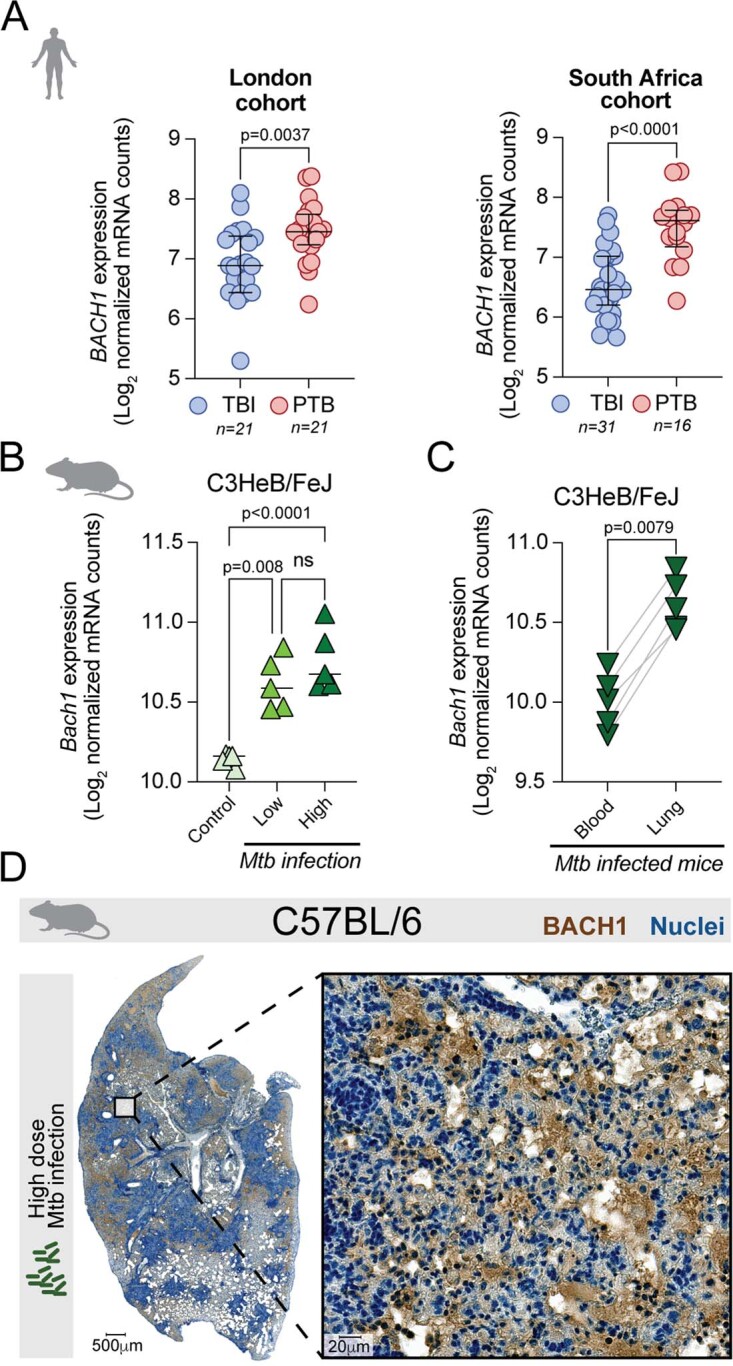
Publicly available data supporting the association of BACH1 expression with TB disease in infected patients and in a murine model of Mtb-induced pulmonary necrosis. RNAseq data from an available data set previously published by Moreira-Teixeira et al. 2020^33^ were re-analyzed. (**A**) *BACH1* mRNA expression was assessed in TBI and PTB patients (TBI (n = 21) vs PTB (n = 21) London cohort, P = 0.0037; TBI (n = 31) vs PTB (n = 16) South Africa cohort P < 0.0001; two-tailed, Mann–Whitney U-test) from two different cohorts (London and South Africa cohorts). Each dot denotes a single patient analyzed and the data shown represent the means ± SEM of the pooled samples for each group. (**B-C**) C3HeB/FeJ mice (n = 5 for each group) were aerosol infected with H37Rv Mtb at different doses (low-dose and high-dose) and RNAseq analysis performed in lung homogenates and blood at 42 p.i. Each symbol represents an individual animal within the group and data shown are from one experiment performed. (**B**) mRNA expression of *Bach1* in the lungs of Mtb-infected and uninfected animals (control vs low, P < 0.0001; control vs high, P = 0.008; one-way ANOVA). (**C**) Paired comparison of *Bach1* mRNA expression in the lungs and blood samples from the same animal (P = 0.0079, two-tailed, Mann–Whitney U-test). (**D**) Representative lung tissue sections from C57BL/6 mice (n = 7) infected with H37Rv Mtb strain at high-dose (~1500-2000 c.f.u) and euthanized 26 days later. The sections were stained for Bach1. Strong staining (brown) for Bach1 was observed in inflamed areas in the lungs from these animals. The images shown are representative of those observed in 7 individual animals from one experiment performed. (*ns*, not significant). Source data

We previously described markedly reduced GSH levels and *GPX4* mRNA expression together with elevated lipid peroxidation in PTB patients^11,13^. One explanation for the failure of this antioxidant response to control excess lipid ROS generation in these patients could be limited NRF2 activation resulting from the induction/activation of its suppressor, BACH1. To test this hypothesis, we analysed *BACH1* mRNA expression in PBMC from TB patients from distinct cohorts in Brazil and South Africa. We first compared *BACH1* mRNA levels in purified peripheral blood CD14^+^ monocytes obtained from Brazilian PTB patients (respiratory symptoms suggestive of TB for more than 2 weeks and culture positive for Mtb (MGIT or solid media)) with patients with no symptoms, normal chest X-ray, but with positive IGRA tests (TBI) and healthy control (HC) individuals. PTB patients exhibited higher *BACH1* mRNA levels compared with TBI and HC patients. While TBI patients showed lower BACH1 mRNA expression than PTB patients, their levels were nevertheless greater than those of HC individuals (Fig. 1a). These findings were supported by publicly available RNA-seq data from an independent study involving two separate cohorts in London and South Africa^33^, in which enhanced *BACH1* mRNA levels were observed in whole blood from PTB compared with TBI patients (Extended Data Fig. 1a). We also evaluated *BACH1* mRNA expression in whole blood obtained from HC patients as well as TB patients classified as incident, subclinical or clinical in a separate cohort in Cape Town, South Africa. Clinical TB patients displayed the highest mRNA expression for *BACH1* when compared with incident TB patients and HC individuals (Fig. 1b). Furthermore, in an adolescent cohort followed longitudinally during disease progression^34^, significant upregulation of *BACH1* in whole blood samples preceded diagnosis of active TB by ~8 months (Fig. 1c). In addition, we compared *BACH1* mRNA expression in different column-purified peripheral blood cell populations (monocytes, neutrophils and T cells) from PTB patients in a separate analysis involving the Brazilian cohort. Interestingly, monocytes from PTB patients showed higher *BACH1* mRNA expression than T cells or neutrophils (Fig. 1d). To further investigate the involvement of BACH1 in TB disease, we examined post-mortem lung tissue sections from Brazilian PTB patients obtained after autopsy. Strong BACH1 staining was observed in necrotic areas (fragmented/faint nuclei cells) compared with inflamed areas (displaying minimal or no dead/dying cells) (Fig. 1e). These findings indicated that *BACH1* expression is highly induced by Mtb infection and increases in blood as well as in necrotic areas in situ during progression to active TB disease.

### Enhanced pulmonary Bach1 expression in Mtb-infected animals

To extend these clinical observations, we assessed Bach1 protein expression in lung tissue sections from rhesus macaques at 15–16 weeks post infection with Mtb^35^. Solid granulomas from these animals showed strong Bach1 staining in the central myeloid cell enriched core compared with cells located at the periphery (cuff) of the lesion (Fig. 1f top). Furthermore, Bach1 staining was observed in necrotic granulomas from these macaques, with the strongest expression occurring in cells surrounding and within the necrotic core (Fig. 1f middle and bottom).

We next assessed the association of Bach1 expression with necrotic pathology in a murine Mtb-infection model known to develop hypernecrotic granulomas following low-dose Mtb infection (B6.Sst1^S^ mice)^36,37^. Supporting our observations in non-human primates (NHP), we again observed increased Bach1 staining in the central portion of granulomatous lesions and reduced staining for the transcription factor in cells located at the periphery as well as in tissue areas surrounding the granuloma (Fig. 1g). Importantly, strong Bach1 staining was observed in cells undergoing necrosis within granulomas (Fig. 1g bottom left), with weaker staining seen in mononuclear cells (probably alveolar macrophages) within the alveolar space (Fig. 1g bottom right). In support of these histopathological findings, analysis of RNA-seq data obtained from publicly available data revealed increased *Bach1* mRNA levels in the lungs of Sst1^S+^C3HeB/FeJ mice following aerosol H37Rv infection (Extended Data Fig. 1b). A paired analysis between samples obtained from blood and lung homogenates of Mtb-infected mice demonstrated elevated Bach1 mRNA expression in lungs compared with cells in the circulation (Extended Data Fig. 1c). Furthermore, pronounced staining for Bach1 was observed in lung sections from conventional C57BL/6 animals (Extended Data Fig. 1d).

Collectively, these findings revealed enhanced necrosis-associated Bach1 expression in pulmonary granulomatous tissue of experimentally infected animals.

### Ablation of Bach1 results in enhanced host resistance to Mtb

**Fig. 2:**
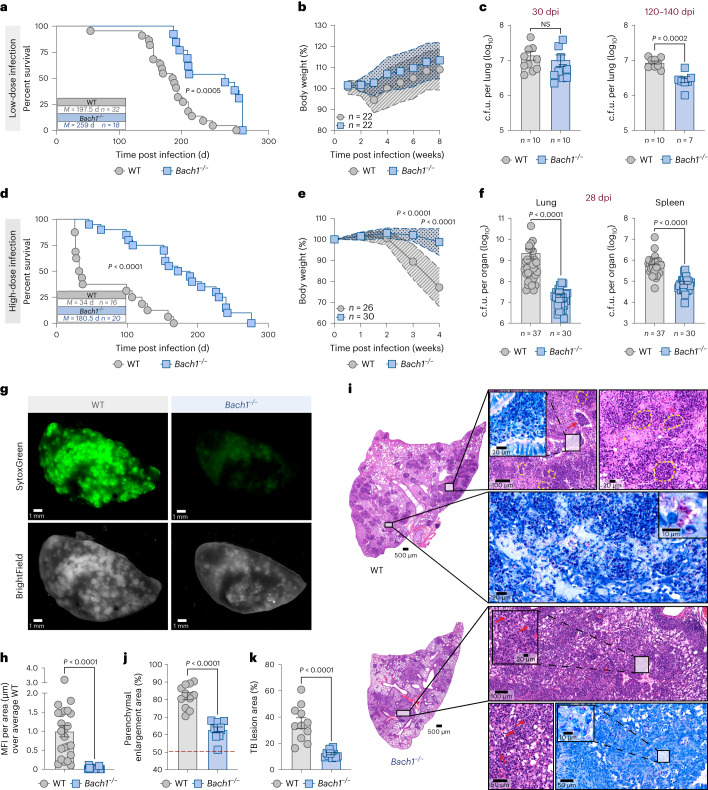
Bach1 deficiency enhances host resistance to experimental Mtb infection correlating with decreased bacterial loads and tissue necrosis. **a**–**i**, *Bach1*^*−/−*^ and WT mice were aerosol infected with H37Rv Mtb at low dose (~100–250 c.f.u; **a**–**c**) or high dose (~1,000–2,000 c.f.u; **d**–**i**) by IPH inoculation as described in Methods. **a**,**d**, Survival curves (WT vs *Bach1*^−/−^ in **a**, *P* = 0.0005; WT vs *Bach1*^−/−^ in **d**, *P* < 0.0001; Mantel–Cox test) and **b**,**e**, body weight of Mtb-infected *Bach1*^−/−^ and WT mice (data pooled from three independent experiments; WT vs *Bach1*^*−/−*^ in **e**, *P* < 0.0001, two-way ANOVA). **c**, Mice infected at low-dose Mtb were euthanized at 30 and 120 dpi and pulmonary bacterial loads were evaluated (data pooled from 2 experiments; WT vs *Bach1*^−/−^ for 120-day time-point, *P* = 0.0002, two-tailed, Mann–Whitney *U*-test). **f**, Bacterial loads in the lungs and spleens of mice infected at high-dose Mtb assayed at 28 dpi (data pooled from 6 separate experiments; WT vs *Bach1*^−/−^ in lungs *P* < 0.0001 and in spleens *P* < 0.0001, two-tailed, Mann–Whitney *U*-test). **g**, Lung necrosis evaluated by SytoxGreen DNA staining. **h**, MFI of SytoxGreen staining per area of whole lung samples. Pooled results of 3 independent experiments are shown (WT, *n* = 23 vs *Bach1*^−/−^ *n* = 18; *P* < 0.0001, two-tailed, Mann–Whitney *U*-test). **i**, Representative hematoxylin-eosin (H&E) (purple) and Ziehl-Neelsen (ZN) (blue) images of lungs isolated from *Bach1*^−/−^ (bottom) and WT mice (top). Each image is representative of tissue sections from at least 5 individual mice per experiment. Mtb-infected WT mice displayed extensive necrotic lesions (yellow dashed line and asterisk) with intrabronchial accumulation of necrotic cellular material (red arrow) presenting elevated numbers of acid-fast bacilli (inset). On the bottom panel, reduced areas of inflammation and necrosis along with few AFB per cell (insets) were observed in lungs of *Bach1*^−/−^ mice. **j**, Parenchymal enlargement (*P* < 0.0001, two-tailed, Mann–Whitney *U*-test) and **k**, TB lesion areas (*P* < 0.0001, two-tailed, Mann–Whitney *U*-test) were measured in the lung sections. **j**,**k**, Pooled results from two independent experiments (*n* = 11 per group). The data shown in **a**–**f**, **h**, **j** and **k** are presented as mean ± s.e.m. of samples. Source data

To assess the functional role of Bach1 in Mtb-induced disease, we examined the outcome of Mtb infection in *Bach1*^−/−^ and WT mice at different doses of infection. When aerosol infected with H37Rv strain at low dose (~150–250 c.f.u.s), *Bach1*^−/−^ mice showed a small but significant increase in resistance to Mtb, with a median survival time of 250 days post infection (dpi) vs 191 d for WT control animals (Fig. 2a). Although no significant differences in initial body weight loss or pulmonary bacterial loads were observed between the animal groups at 30 dpi (Fig. 2b,c), *Bach1*^−/−^ mice displayed lung Mtb numbers that were lower than those of their WT counterparts by 120–140 dpi (Fig. 2c).

In contrast, when infected with H37Rv at high dose (~1,500–2,000 c.f.u), a condition routinely used to enhance lung necrosis, *Bach1*^−/−^ mice exhibited markedly enhanced resistance to Mtb, succumbing almost 4 months later than infected WT control animals (median survival time of 180.5 dpi for *Bach1*^−/−^ mice vs 34 dpi for WT animals) (Fig. 2d). This augmented host survival was reflected in a striking protection against body weight loss (Fig. 2e) as well as greatly reduced pulmonary and splenic bacterial loads in the *Bach1*^−/−^ mice measured at 28 dpi (Fig. 2f).

To directly assess tissue necrosis in Mtb-infected animals, we injected mice intravenously with SytoxGreen dye before euthanasia, an approach that detects bioavailable DNA in dying cells as well as the extracellular tissue matrix^11^. When examined by fluorescence microscopy, whole lungs from Mtb-infected WT mice showed extensive areas of SytoxGreen staining that were markedly diminished in infected *Bach1*^−/−^ animals (Fig. 2g). A similar reduction was observed in the quantitative SytoxGreen mean fluorescence intensity (MFI) of the whole lung images (Fig. 2h). More detailed histopathological examination of the lung sections from Bach1-deficient mice revealed a pronounced protection against tissue damage as evidenced by limited cellular necrosis and cellular debris within bronchial as well as alveolar spaces (Fig. 2i). Furthermore, we observed lowered numbers of acid-fast bacilli (AFB) per cell in lungs of mice deficient in Bach1 compared with similarly stained sections from WT animals (Fig. 2i, inserts). Additional quantitative analysis of the lung sections revealed a marked reduction in the size of the parenchymal areas as well as Mtb-infected granulomatous tissue in the *Bach1*^−/−^ animals compared with the corresponding tissue in WT mice (Fig. 2j,k, respectively).

**Fig. 3:**
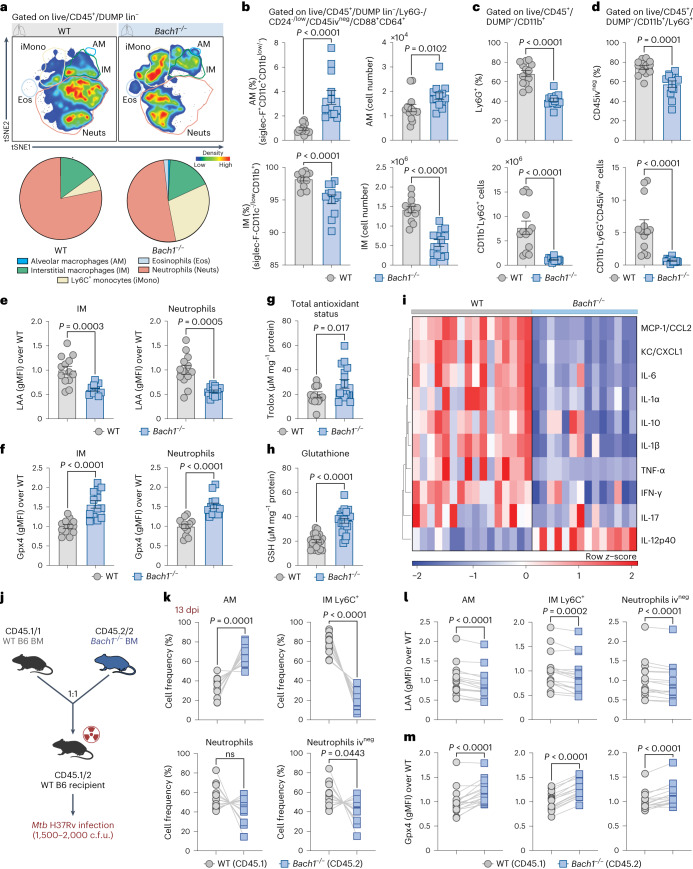
The effects of Bach1 expression on pulmonary inflammation is reflected in the modulation of the cell-intrinsic oxidative response. **a**–**i**, *Bach1*^−/−^ and WT mice were infected at high-dose Mtb and euthanized at 28 dpi. **a**, tSNE analysis of myeloid cells in the lungs of Mtb-infected mice (*n* = 5 per group). **b**, Frequencies and numbers of AM (Live/CD45^+^/DUMP^-^/Ly6G^−^/CD45iv^neg^/CD88^+^CD64^+^/CD11b^−/low^/CD11c^+^/Siglec-F^+^), IM (Live/CD45^+^/DUMP^−^/Ly6G^−^/CD45iv^neg^/CD88^+^/CD64^+^/CD11b^hi^/CD11c^-/low^/Siglec-F^−^) (two-tailed, Mann–Whitney *U*-test), **c**, total neutrophils (Live/CD45^+^/DUMP^−^/CD11b^+^Ly6G^+^) (two-tailed Mann–Whitney *U*-test) and **d**, parenchymal neutrophils (Live/CD45^+^/DUMP^−^/CD11b^+^Ly6G^+^/CD45iv^−^) (two-tailed Mann–Whitney *U*-test) were enumerated; DUMP: TCRβ, TCRγδ, NK1.1, B220. **e**, Lipid peroxidation (LAA staining) (two-tailed Mann–Whitney *U*-test) and **f**, Gpx4 levels (over the average MFI of the WT) (two-tailed Mann–Whitney *U*-test) in live IM and neutrophils. **b**–**f**, Pooled data from 3 independent experiments are shown (WT *n* = 13 vs *Bach1*^−/−^ *n* = 12). **g**, Total antioxidant status (*n* = 16 per group, pooled from 3 experiments; two-tailed Mann–Whitney *U*-test). **h**, Glutathione measured in lung homogenates (WT *n* = 20 vs *Bach1*^−/−^ *n* = 22, pooled from 4 experiments; two-tailed Mann–Whitney *U*-test). **i**, Heatmap visualization of 10 cytokines/chemokines measured in lung homogenates. Pooled data from 3 independent experiments are shown (WT *n* = 16 vs *Bach1*^−/−^ *n* = 14). **j**, Schematic of mixed BM chimaeric protocol. Mice were euthanized at 13 dpi. **k**, Analysis of cell frequency (two-tailed Wilcoxon-matched pairs), **l**, lipid peroxidation (two-tailed Wilcoxon-matched pairs) and **m**, Gpx4 expression (two-tailed Wilcoxon-matched pairs) in AM, IM and neutrophil populations in the lungs of Mtb-infected mixed (WT/*Bach1*^−/−^) BM chimaeras. Results shown are pooled from 2 independent experiments (*n* = 15 per group; Wilcoxon-matched pairs). Data shown in **b**–**m** are presented as mean ± s.e.m. and each symbol represents an individual animal. Source data

**Extended Data Fig. 2:**
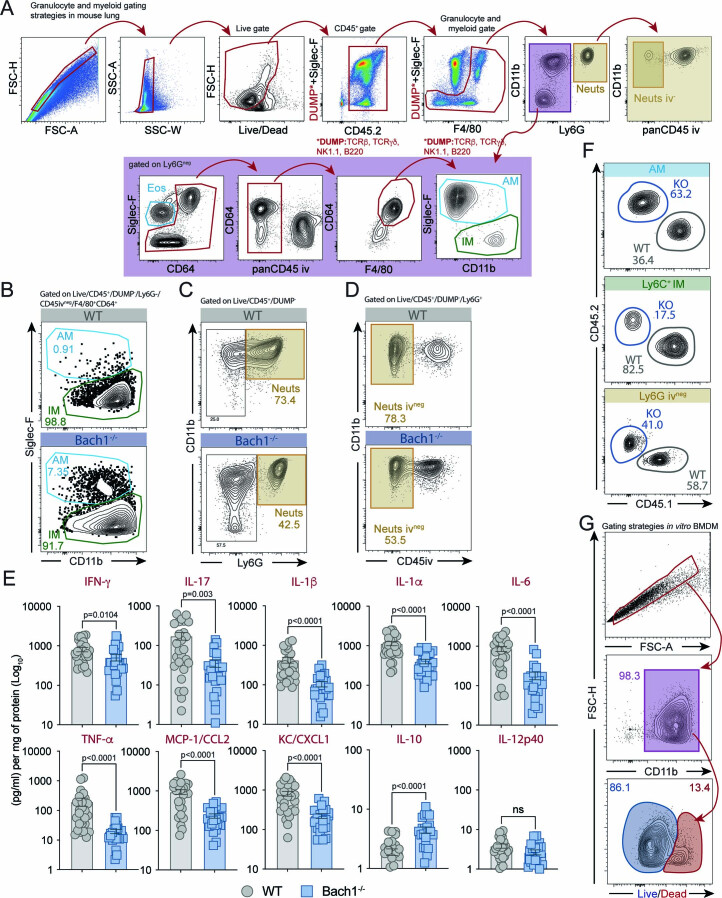
Gating strategies for identifying granulocyte and myeloid cells by flow cytometry as well as cytokine/chemokine production in lungs. Single-cell suspensions were prepared from the lungs of naïve animals i.v. injected with labeled panCD45 antibody prior euthanasia to identify cells located within the lung vasculature. Gating strategy was used to identify myeloid populations in Mtb-infected lungs. *DUMP: TCRβ, TCRγδ, NK1.1, B220; AM, alveolar macrophages, IM, interstitial macrophages; Eos, eosinophils; Neuts, neutrophils; Neuts iv-, parenchymal neutrophils. Anti-Siglec-F antibody was added to the panel in the same channel as the DUMP makers and analyzed in combination with F4/80 to isolate AM and EOS out from DUMP+ cells as showed in the FACS plot. (**B-D**) Sample FACS plot of (**B**) parenchymal macrophages gated on Live/CD45^+^/DUMP^−^/Ly6G^−^/CD45iv^neg^/F4/80^+^CD64^+^ events, (**C**) total neutrophils (gated on Live/CD45^+^/DUMP^−^/CD11b^+^Ly6G^+^) and (**D**) parenchymal neutrophils (gated on Live/CD45^+^/DUMP^−^/CD11b^+^Ly6G^+^/CD45iv^neg^) shown in the Fig. 3b,c. **(E)** Summary data of cytokine/chemokine levels in lung homogenates from Mtb^−^infected *Bach1*^*-/-*^ (n = 23) and WT (n = 26) at 28 days p.i. (pooled from five independent experiments; two-tailed, Mann–Whitney U-test). (**F**) Sample FACS plot of CD45.1 (WT) vs CD45.2 (KO) within AM, Ly6C^+^ IM, total and parenchymal neutrophils shown in the Fig. 3k–m. (**G**) Sample gating strategy used for the *in vitro* experiments with BMDMs. FACS plots shown in **A**-**D** and **F**-**G** are representative of at least two independent experiments performed. Each symbol represents an individual animal within the group. Significant differences are indicated with asterisks (*ns*, not significant). Source data

**Extended Data Fig. 3:**
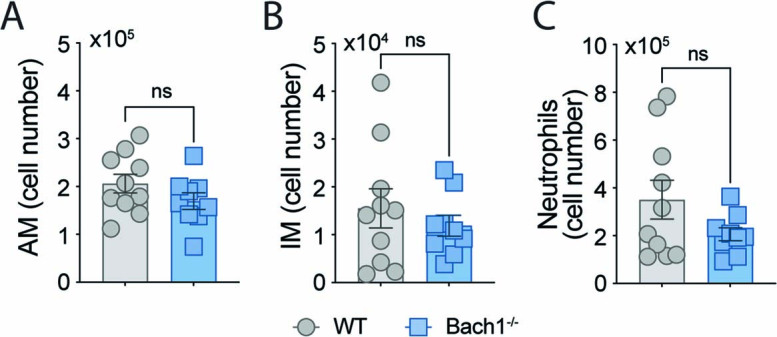
Bach1 deficiency does not affect numbers of myeloid cells in the lungs of mice at baseline. (**A**–**C**) Total numbers of (A) AMs (Live/CD45iv^neg^/F4/80^+^CD64^+^/CD11b^-^/CD11c^+^/Siglec-F^+^), (**B**) IMs (Live/CD45iv^neg^/ F4/80^+^CD64^+^/CD11b^hi^/CD11c^-/l^°^w^/Siglec-F^-^), and (**C**) neutrophils (Live/CD45^+^/DUMP^−^/CD11b^+^Ly6G^+^) in the lungs of uninfected *Bach1*^-/-^ and WT mice as measured by flow cytometry. Results are pooled from of three independent experiments performed (WT n = 10 vs *Bach1*^*-/-*^ n = 9) and the data shown are presented as mean values ± SEM of the pooled samples for each group. Each symbol represents an individual animal from each group. Statistical significance was assessed by the Mann-Whitney test, two-tailed. (*ns*, not significant). Source data

Flow cytometric analysis performed on single-cell lung suspensions revealed a significant difference in the cellular composition of the myeloid compartment in the two groups of animals (Fig. 3a–d and Extended Data Fig. 2a). Notably, t-distributed stochastic neighbour embedding (tSNE) analysis showed diminished enrichment of neutrophils (Live/CD11b^+^Ly6G^+^) in lung parenchyma of Bach1-deficient mice consistent with the overall decrease in tissue pathology (Fig. 3a,d). Interestingly, infected *Bach1*^−/−^ mice displayed increased frequencies and numbers of AM (Live/CD45iv^neg^/F4/80^+^CD64^+^/CD11b^−/low^/CD11c^+^/Siglec-F^+^), suggesting a role for Bach1 in regulating the survival of these cells (Fig. 3a,b and Extended Data Fig. 2b). This change was associated with a corresponding pronounced reduction in both the frequency and numbers of interstitial macrophages (IM; Live/CD45iv^neg^/F4/80^+^CD64^+^/CD11b^hi^/CD11c^−/lo^^w^/Siglec-F^-^) (Fig. 3a,b and Extended Data Fig. 2b) and neutrophils in the Bach1-deficient animals (Fig. 3d and Extended Data Fig. 3c,d). Of note, Bach1 deficiency did not affect the baseline numbers of pulmonary AM, IM and neutrophils in uninfected animals (Extended Data Fig. 3).

Lipid peroxidation is a hallmark of oxidative stress-mediated ferroptotic cell death and the presence of GSH as a co-factor for Gpx4 is critical for the detoxification process that protects host cell membranes^16^. Since Bach1 is a known suppressor of Nrf2, a transcription factor essential for the induction of a number of antioxidant genes^24^, we hypothesized that Bach1 expression influences host resistance to Mtb infection by modulating the host antioxidant response. To investigate whether Bach1 modulates host redox responses, we measured lipid peroxidation, Gpx4 expression, total antioxidant status (TAS) as well as GSH levels in whole lungs. As predicted, the lungs of the Mtb-infected Bach1-deficient mice showed decreased lipid peroxidation staining along with increased expression of Gpx4 in both IM and neutrophils as measured by flow cytometry (Fig. 3e,f). In addition, deficiency in Bach1 was associated with augmented pulmonary TAS and GSH levels (Fig. 3g,h). Consistent with the elevated host antioxidant response and general decrease in tissue inflammation, *Bach1*^−/−^ mice displayed a profound reduction in the levels of inflammatory cytokines (except for IL-12p40) and chemokines when compared with infected WT animals (Fig. 3i and Extended Data Fig. 2e).

Together, the above findings demonstrate a detrimental role for Bach1 in regulating host susceptibility to Mtb infection that is closely associated with oxidative stress responses, pulmonary tissue damage and inflammation.

### Bach1 promotes cell-intrinsic host susceptibility to Mtb

As described above, mice deficient in Bach1 displayed enhanced control of bacterial burden and lung tissue damage associated with increased frequencies of AM along with reduced percentages of parenchymal inflammatory macrophages and neutrophils. To investigate the cell-intrinsic requirement for Bach1 expression in AM, IM and neutrophils, we generated mixed bone-marrow (BM) chimaeric mice by adoptive transfer of a 1:1 mixture of BM cells from *Bach1*^−/−^ and WT distinguishable by CD45 allelism (*Bach1*^−/−^ (CD45.2^+^) and WT (CD45.1^+^)). After complete reconstitution (~3 months post BM transfer), mice were infected with Mtb at high dose and single-cell flow cytometric analysis was performed at 13 dpi (Fig. 3j). Interestingly, we found that *Bach1*^−/−^ CD45.2^+^ AM were enriched in the lungs of the Mtb-infected chimaeric mice when compared with the frequency of WT CD45.1^+^ AM. In contrast, the proportion of inflammatory monocyte-derived macrophages (Ly6C^+^ IM) and neutrophils staining for CD45.2^+^ in the lung parenchyma was diminished in these animals (Fig. 3k and Extended Data Fig. 2f). Furthermore, AM, Ly6C^+^ IM and parenchymal neutrophils deficient in Bach1 displayed reduced lipid peroxidation (Fig. 3l) along with enhanced Gpx4 expression (Fig. 3m) compared with WT cells in the same lung tissue. Taken together, these mixed BM chimaera experiments demonstrate that Bach1 expression in AM, inflammatory monocyte-derived macrophages and neutrophils modulates cell-intrinsic host oxidative stress in the pulmonary response to Mtb infection.

### Bach1 regulates ferroptosis-related gene expression in vivo

**Fig. 4:**
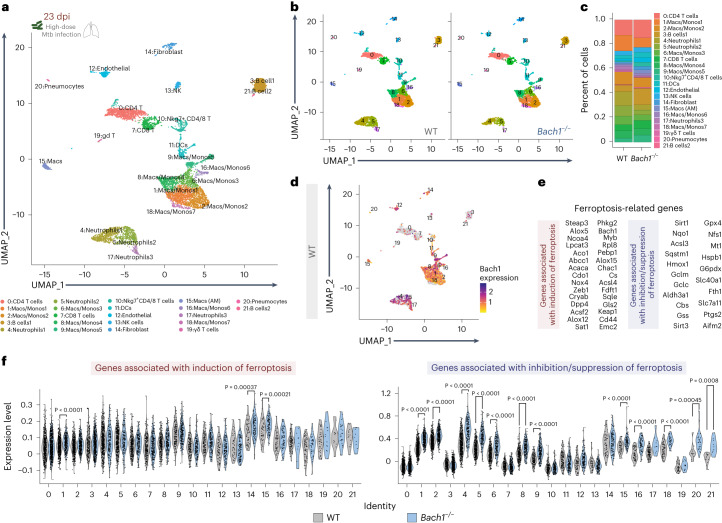
Pulmonary cell populations from Bach1-deficient mice display enriched expression of genes associated with inhibition/suppression of ferroptosis (FRG). *Bach1*^−/−^ and WT mice were infected at high dose with Mtb. Mice were euthanized at 23 dpi and single-cell suspensions from lungs were prepared for analysis (*n* = 5 per group, samples pooled from 1 experiment). **a**, UMAP plot representing the clustering pattern of cells from scRNA-seq data of single-cell suspensions from lungs of Mtb-infected animals. Each dot denotes a single cell and is coloured on the basis of the automated cluster identification. Clusters of cells belonging to a certain cell type are demarcated and indicated on the plot. **b**, UMAP comparing cells from Mtb-infected WT (left) and *Bach1*^−/−^ (right) mice lungs. **c**, Percent of cells from each cluster found in Mtb-infected *Bach1*^−/−^ and WT mice. **d**, UMAP plot showing Bach1 expression in the cluster of cells identified. **e**, List of FRG used to assess expression of genes associated with the induction (left) or inhibition/suppression (right) of ferroptotic cell death. **f**, Violin plot of FRG expression in different cell clusters in *Bach1*^−/−^ and WT mice. The statistical differences between WT and *Bach1*^−/−^ mice for each cluster were calculated using two-tailed Wilcoxon test, with cut-off at *P* < 0.001. Source data

**Extended Data Fig. 4:**
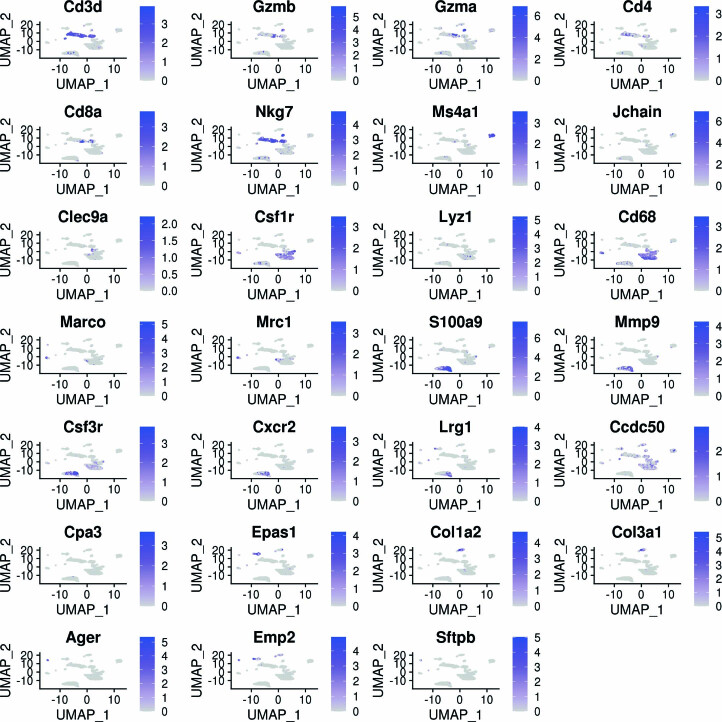
UMAP showing the expression of genes used to identify the different cell population clusters in the scRNA-seq dataset. Expression levels of cell type defining markers are shown as blue dots overlapping the original UMAP used to determine the clusters of cells (n = 5, each group; samples pooled from one experiment). Source data

**Extended Data Fig. 5:**
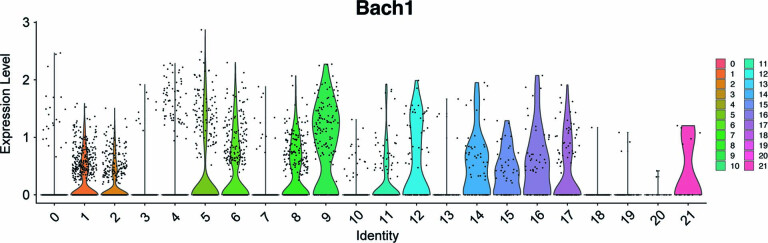
Bach1 expression in different cell population clusters in the scRNA-seq dataset. Violin plot of Bach1 expression in different cell clusters in *Bach1*^*-/-*^ and WT mice. (n = 5, each group; samples pooled from one experiment). Source data

**Extended Data Fig. 6:**
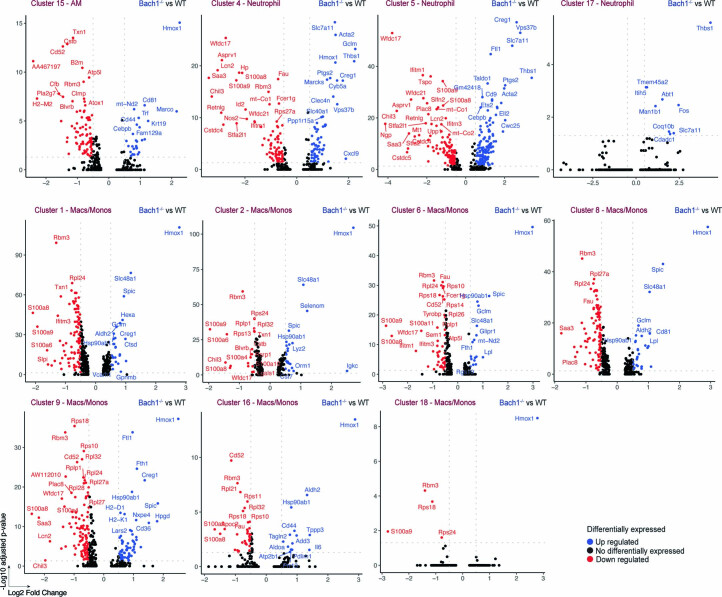
Bach1-deficient mice exhibit increased expression of antioxidant genes associated with iron and glutathione metabolism. Volcano plot of the differentially expressed genes between *Bach1*^-/-^ and WT AM (cluster 15), neutrophil (clusters 4, 5 and 17) and macrophage/monocyte populations (cluster 1, 2, 6, 8, 9, 16, 18) are shown (n = 5, each group; samples pooled from one experiment). Vertical and horizontal red lines indicate log fold change ≥ 0.5 and p-ad value < 0.05 respectively. Positive-fold change indicates higher expression in *Bach1*^-/-^ relative to WT. Statistical significance in was calculated with the Wilcoxon Rank-Sum test. Source data

To investigate the cellular mechanism of Bach1-driven Mtb susceptibility, single-cell RNA sequencing (scRNA-seq) was performed on single-cell suspensions generated from lungs of Mtb-infected *Bach1*^−/−^ and WT mice at 23 dpi. Uniform manifold approximation and projection (UMAP) display of Seurat clustering shows 22 cell population clusters that include multiple myeloid cells, lymphocytes (B cell, T cell, γδT cells and NK cells), pneumocytes, endothelial cells and fibroblasts (Fig. 4a and Extended Data Fig. 4). While each cluster was represented in the WT and *Bach1*^−/−^ mice datasets, the relative frequency of each population was distinct between groups (Fig. 4b,c). Specifically, *Bach1*^−/−^ mice displayed a marked enrichment of AM along with a lowered fraction of both neutrophil (clusters 4, 5 and 17) and macrophage/monocyte populations (clusters 1, 2, 6, 8, 9, 16 and 18) in comparison with WT animals (Fig. 4b,c). Of note, Bach1 expression was found to be elevated mainly in AM, neutrophil and macrophage/monocyte populations compared with other immune cells (Fig. 4d and Extended Data Fig. 5). Importantly, these cell populations in *Bach1*^−/−^ mice exhibited upregulation of antioxidant genes associated with both GSH (for example, *Gclm, Slc7a11*) and iron metabolism (for example, *Hmox1*, *Fth1*, *Spic*) (Extended Data Fig. 6), supporting our data demonstrating elevated levels of GSH and TAS in whole lungs of Bach1-deficient animals (Fig. 3g,h).

Since the iron and GSH metabolic pathways are both strongly associated with the modulation of ferroptotic cell death, we next investigated the expression of ferroptosis-related genes (FRG) in each identified cell cluster. We chose 51 genes (Supplementary Table 3) that were previously reported to be involved in ferroptosis, either promoting or preventing the ferroptotic cell death pathway^38,39^. We stratified FRG on the basis of their known functions into genes associated with induction of ferroptosis and genes associated with inhibition/suppression of this necrotic cell death modality (Fig. 4e,f). Interestingly, Mtb infection triggered FRG associated with the induction of ferroptosis (pro-oxidative genes) at similar levels in all cell populations identified by scRNA-seq analysis in both *Bach1*^−/−^ and WT mice. However, when FRGs associated with inhibition/suppression of ferroptosis (for example, antioxidant genes) were analysed, we found that Bach1 deficiency was accompanied by an enrichment of these FRGs in myeloid cells (neutrophils, AM and monocyte-derived macrophage/monocytes), as well as in endothelial cells and pneumocytes. No major change in FRGs was observed in clusters associated with lymphocytes (clusters 0, 3, 7, 10, 13 and 19), dendritic cells (DCs) (cluster 11) and fibroblasts (cluster 14). Collectively, these findings reveal an important role for Bach1 in regulating genes associated with the ferroptotic cell death pathway in distinct cell populations in response to Mtb infection.

### Bach1 regulates Mtb-induced macrophage necrosis in vitro

**Extended Data Fig. 7:**
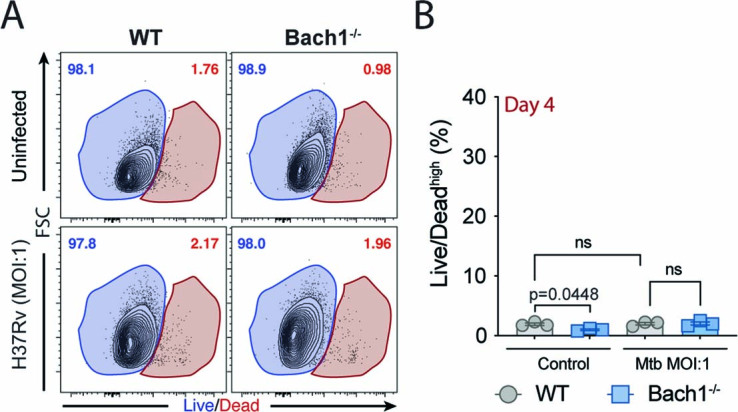
Cell death measurement of BMDM infected with Mtb at low MOI. *Bach1*^-/-^ and WT BMDMs were infected with H37Rv Mtb at an MOI of 1. (**A**) Sample FACS plots demonstrating Mtb-induced macrophage necrosis *in vitro* as measured by Live/Dead staining on the X axis at day 4 p.i. (**B**) Summary graph of data shown in A. The data represent means ± SEM of triplicate samples and are representative of two independent experiments. Statistical significance was assessed by one-way ANOVA for the indicated experimental conditions. (ns, not significant). Source data

**Extended Data Fig. 8:**
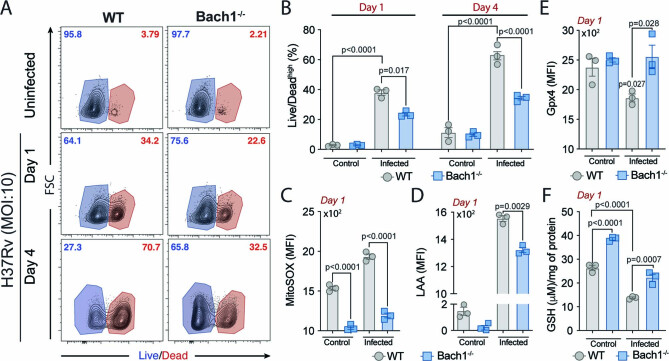
Bach1 deficiency suppresses Mtb-induced macrophage necrosis by enhancing the cellular antioxidant response. **A-F**) *Bach1*^-/-^ and WT BMDMs were infected with H37Rv Mtb at an MOI of 10. (**A**) Sample FACS plots demonstrating Mtb-induced macrophage necrosis *in vitro* as measured by Live/Dead staining on the X axis at day 1 versus day 4 p.i. (**B**) Summary graph of data shown in A presenting the means ± SEM of triplicate samples analyzed. (**C**) Mitochondrial superoxide (measured by MitoSOX staining), (**D**) lipid peroxidation (evaluated by LAA staining) and (**E**) Gpx4 protein expression analyzed in live CD11b^+^ cells by flow cytometry. (**F**) Intracellular GSH levels measured on day 1 p.i. The data represent means ± SEM of triplicate samples and are representative of at least two independent experiments. Statistical significance was assessed by one-way ANOVA for the indicated experimental conditions. Source data

**Extended Data Fig. 9:**
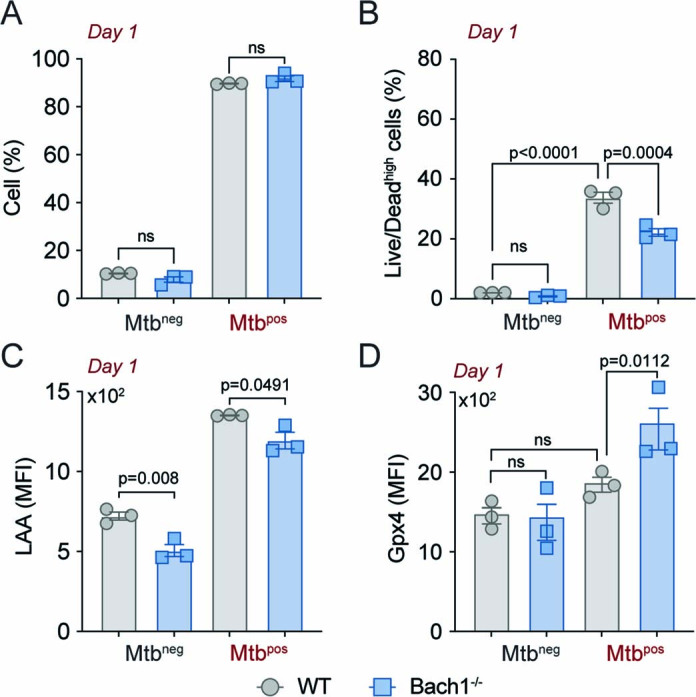
Evaluation of cell death in Mtb-infected and bystander BMDM at an MOI of 10. *Bach1*^*-/-*^ and WT BMDMs were infected with RFP-labeled H37Rv (H37Rv-RFP) Mtb at an MOI of 10 and cell death, lipid peroxidation and Gpx4 expression assessed at day 1 p.i. (**A**) Frequencies of Mtb-infected (Mtb^p^°^s^) and bystander (Mtb^neg^) BMDMs. (**B**) Frequency of Live/Dead^high^ cells within total CD11b^+^ cells. (**C**) Lipid peroxidation (evaluated by LAA staining) and (**D**) Gpx4 protein expression analyzed by flow cytometry. The data represent means ± SEM of triplicate samples and are representative of two independent experiments. Statistical significance was assessed by one-way ANOVA for the indicated experimental conditions. (ns, not significant). Source data

We previously showed that Mtb-infected macrophages undergo ferroptotic cell death^11^ and further demonstrated that Gpx4 expression/activity negatively regulates this process^13^. Bach1 has been reported to induce ferroptosis^24^ and as shown above, Bach1-deficient pulmonary macrophages overexpress genes associated with inhibition/suppression of this form of death. To evaluate whether Bach1 plays a role in promoting necrotic cell death in Mtb infection in vitro, we generated bone marrow-derived macrophages (BMDM) from *Bach1*^−/−^ and WT mice and infected them with H37Rv at a multiplicity of infection (MOI) of 10, since consistent with previous findings, we found no significant difference in macrophage death upon Mtb infection at a low MOI of 1 (Extended Data Fig. 7). We observed that Bach1-deficient macrophages were partially resistant to Mtb-induced necrosis as measured by flow cytometry (Extended Data Fig. 8a,b). This protection was associated with reduced levels of mitochondrial superoxide (MitoSOX) and lipid peroxidation, as well as increased Gpx4 expression and augmented intracellular GSH levels (Extended Data Fig. 8c–f). In addition, the resistance of *Bach1*^−/−^ macrophages to cellular necrosis was more pronounced in Mtb-infected than bystander cells at 1 dpi (Extended Data Fig. 9). The above findings further support a role for Bach1 as a regulator of Mtb-induced necrotic cell death through its effects on the oxidative stress response.

### Bach1 ablation enhances antioxidant levels in B6.Sst1^S^ mice

**Fig. 5:**
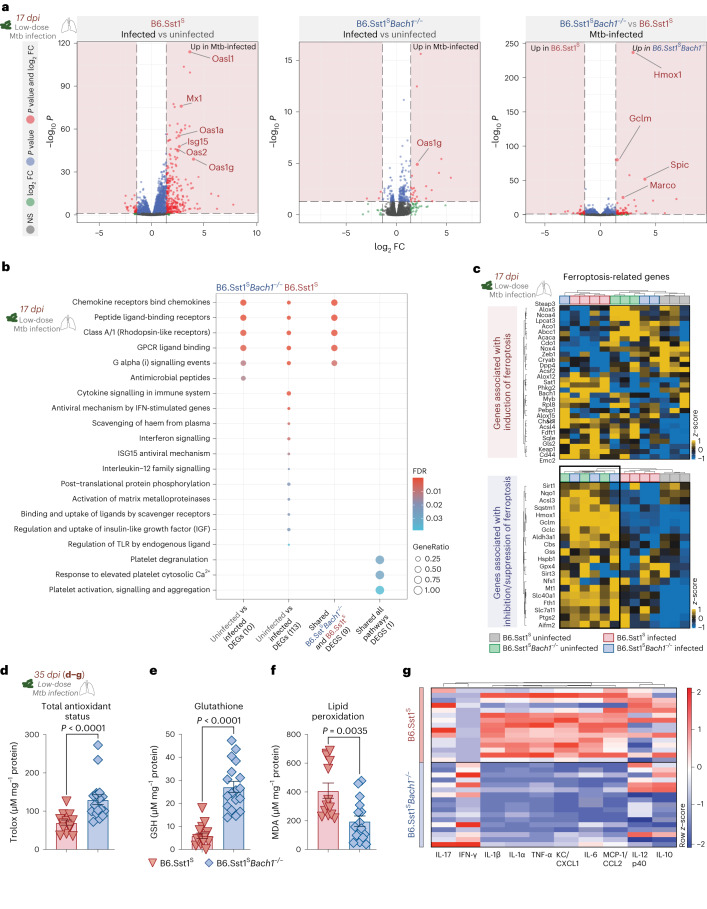
Bach1 deficiency in the hypernecrotic B6.Sst1^S^ murine model of Mtb infection enhances host antioxidant and reduces lung pro-inflammatory responses. **a**–**c**, B6.Sst1^S^ and B6.Sst1^S^*Bach1*^−/−^ mice were infected at low dose with Mtb. Mice were euthanized at 17 dpi and single-cell suspensions from lungs were isolated from Mtb-infected and uninfected animals for RNA-seq analysis (*n* = 3–4 per group, samples obtained from 1 experiment). **a**, Volcano plots from DEGs identified. The comparison betweengroups is indicated. Differentially expressed genes are represented in red, genes with log_2_FC > 1.4 and <- 1.4 are in green, genes with FDR < 0.05 are in blue and genes that are not significant are in grey. **b**, Enrichment analysis plots displaying the augmented pathways from the identified DEGs in the comparisons as indicated. Dot sizes represent the gene ratio in the pathway, fill colours are the FDR values. **c**, Heat map of FRG DEGs associated with induction (top) and suppression (bottom) of ferroptosis identified in the Mtb-infected and uninfected B6.Sst1^S^ and B6.Sst1^S^*Bach1*^−/−^ mice. Coloured row at the top of each panel corresponds to groups of mice analysed. **d**, Total antioxidant status (B6.Sst1^S^ *n* = 14 vs B6.Sst1^S^*Bach1*^−/−^ *n* = 17; two-tailed Mann–Whitney *U*-test), **e**, GSH (B6.Sst1^S^ *n* = 15 vs B6.Sst1^S^*Bach1*^−/−^ *n* = 16; two-tailed Mann–Whitney *U*-test) and **f**, MDA levels (B6.Sst1^S^ *n* = 13 vs B6.Sst1^S^*Bach1*^−/−^ *n* = 16; two-tailed Mann–Whitney *U*-test) measured in lung homogenates from Mtb-infected mice at 35 dpi. Pooled data from 3 independent experiments are shown and each dot denotes an individual animal. **g**, Heat map visualization of 10 cytokines/chemokines measured in lung homogenates from Mtb-infected B6.Sst1^S^ (*n* = 15) and B6.Sst1^S^*Bach1*^−/−^ (*n* = 17) mice (pooled from 3 independent experiments). Data shown in **d**–**f** are presented as mean ± s.e.m. Statistical significance was assessed using the Mann–Whitney test for the indicated experimental conditions. Source data

**Extended Data Fig. 10:**
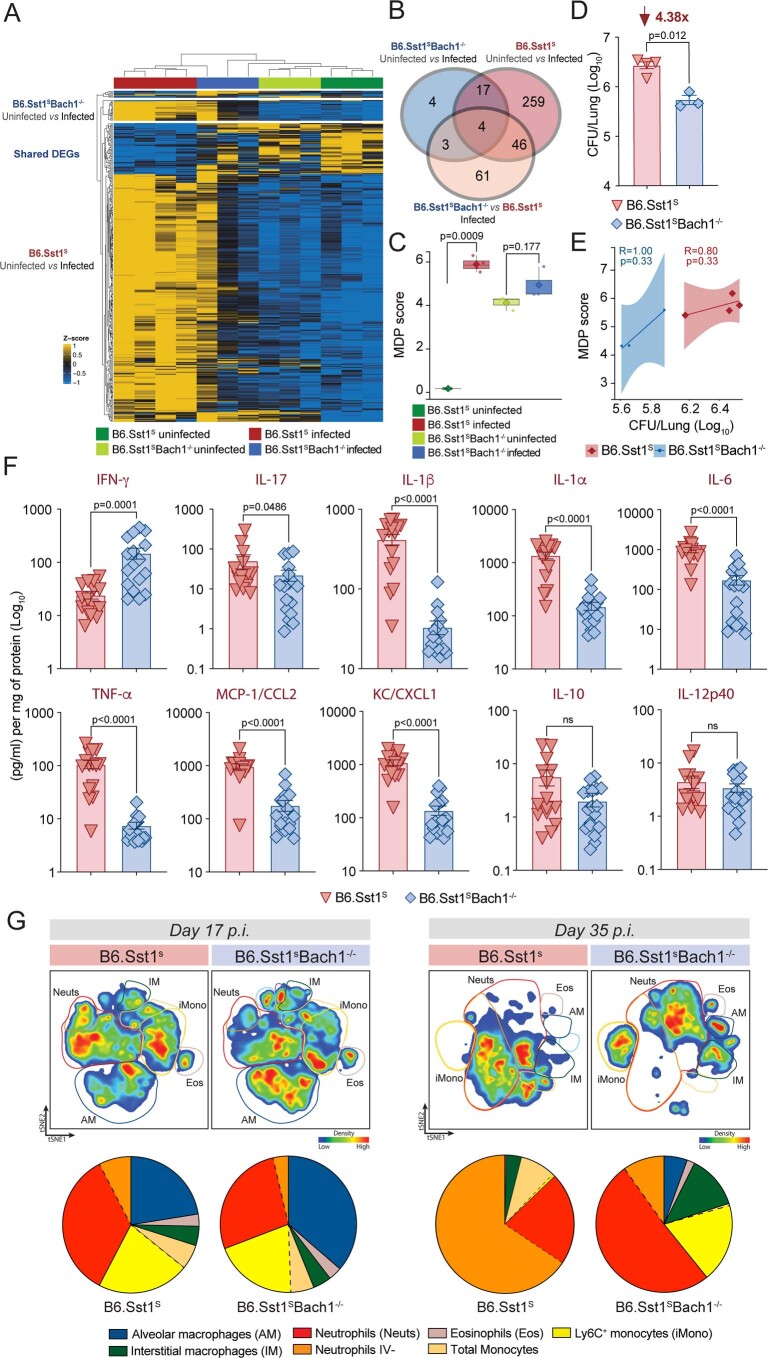
Transcriptional and inflammatory profiles of the lungs in B6.Sst1^S^ and B6.Sst1^S^*Bach1*^*-/-*^ mice. **(A-E)** B6.Sst1^S^ and B6.Sst1^S^*Bach1*^-/-^ mice were infected at low-dose with Mtb. Mice were euthanized at 17 days p.i. and single-cell suspensions from lungs were isolated from Mtb-infected and uninfected animals for RNAseq analysis (n = 3-4, each group; samples from one experiment). (**A**) Heatmap displaying the DEGs identified for each group analyzed. (**B**) Venn diagram plot from the identified DEGs with the corresponding comparisons. (**C**) Molecular degree of perturbation (MDP) plot displaying values as bars for each group. Boxplots shows median (centre line), upper and lower quartiles (box limits) and the interquartile range (whiskers). Each symbol represents an individual animal within the group (n = 3-4, each group) and data shown are from one experiment performed. (**D**) Bacterial loads in the lungs from B6.Sst1^S^ and B6.Sst1^S^*Bach1*^-/-^ mice at 17 days p.i. (**E**) Correlation between MDP values and bacterial counts in the lung from Scatter plot summarizing the MDP and CFU values from B6.Sst1^S^ and B6.Sst1^S^*Bach1*^-/-^ mice at 17 days p.i. (**F**) Summary data of cytokine/chemokine levels in lung homogenates from B6.Sst1^S^ and B6.Sst1^S^*Bach1*^-/-^ mice at 35 days p.i. shown in the Fig. 5G. (B6.Sst1^S^ n = 15 vs B6.Sst1^S^*Bach1*^-/-^ n = 17, pooled from three independent experiments). (**G**) tSNE analysis of the myeloid cells in the lungs of Mtb infected mice at 17 and 35 days p.i. Proportional events from each animal were concatenated (n = 4-5 each group; samples from one experiment). AM (dark blue gate), IM (green gate), neutrophils (red gate), parenchymal neutrophils (orange gate), DCs (purple and light blue gate) and inflammatory monocytes (yellow gate) are indicated in the tSNE plot and the proportion of each cell population cells is shown (bottom). The data shown in D-F are presented as mean values ± SEM of the pooled samples for each group. Statistical significance was assessed by two-tailed, Mann–Whitney test for the indicated experimental conditions. (ns, not significant). Source data

Hypernecrotic granulomas are a hallmark of the severe disease seen in low-dose Mtb-infected C57BL/6 mice carrying the Sst1-susceptible genotype from the C3HeB/FeJ strain (B6.Sst1^S^ mice)^36,37^. To investigate the impact of Bach1 expression on the regulation of host response in this animal model, RNA-seq analysis was performed on pulmonary single-cell suspensions from B6.Sst1^S^ and B6.Sst1^S^*Bach1*^−/−^ mice at 17 dpi in comparison with cells from their respective naïve controls (Fig. 5 and Extended Data Fig. 10).

Differential expression analysis revealed distinct transcriptional profiles between the groups examined (Extended Data Fig. 10a). Venn diagrams demonstrated that Mtb infection specifically triggered 259 differentially expressed genes (DEGs) in control B6.Sst1^S^ mice, while 4 DEGs were uniquely seen in B6.Sst1^S^*Bach1*^−/−^ mice and 61 DEGs were shared between Mtb-infected B6.Sst1^S^ and B6.Sst1^S^*Bach1*^−/−^ mice (Extended Data Fig. 10b). Interestingly, Mtb infection triggered a highly significant molecular degree of perturbation (MDP) in B6.Sst1^S^ but not in B6.Sst1^S^*Bach1*^−/−^ mice (Extended Data Fig. 10c). Of note, the distinct pattern in molecular degree of perturbation analysis seen in B6.Sst1^S^ and B6.Sst1^S^*Bach1*^−/−^ mice did not correlate with the different lung bacterial burdens in the two sets of animals (Extended Data Fig. 10d,e). We also found that Mtb infection triggered higher levels of interferon-stimulated genes (ISG), such as *Oasl1, Mx1* and *Oas1g*, in B6.Sst1^S^ mice than in B6.Sst1^S^*Bach1*^−/−^ animals. Importantly, this analysis revealed that genes related to iron (for example, *Hmox1*, *Spic*) and GSH (for example, *Glcm*) metabolism were upregulated in Mtb-infected Bach1-deficient mice in comparison with Mtb-infected control animals (Fig. 5a). Further examination of the gene set enrichment pathways revealed that Mtb infection triggered a number of pathways related to extensive tissue inflammation in B6.Sst1^S^ mice that are poorly represented in infected B6.Sst1^S^*Bach1*^−/−^ animals. These include pathways associated with IFN-stimulated antiviral mechanisms, haem uptake and matrix metalloproteinase activation. In the opposite direction, the pathway related to antimicrobial peptides was exclusively induced in B6.Sst1^S^*Bach1*^−/−^ mice (Fig. 5b). We next investigated the regulation of genes previously reported to be associated with induction or inhibition/suppression of the ferroptotic cell death pathway in these animals. While both Mtb-infected B6.Sst1^S^ and B6.Sst1^S^*Bach1*^−/−^ mice displayed similar expression of genes associated with ferroptosis induction, Bach1-deficient animals showed increased expression of genes associated with inhibition/suppression of this pathway (Fig. 5c).

To confirm that the above transcriptional changes associated with Bach1 deficiency in this hypernecrotic murine model of pulmonary Mtb infection are reflected in the host oxidative stress response, we measured TAS, GSH and lipid peroxidation in lung homogenates from Mtb-infected B6.Sst1^S^ and B6.Sst1^S^*Bach1*^−/−^ mice at 35 dpi. As predicted, Bach1-deficient mice displayed elevated levels of TAS (Fig. 5d) and GSH (Fig. 5e) along with lowered levels of lipid peroxidation (MDA) (Fig. 5f). Moreover, multiplex protein analysis of lung tissue homogenates from B6.Sst1^S^*Bach1*^−/−^ mice showed a profound reduction in the levels of many cytokines and chemokines typically associated with the inflammatory response, such as IL-1β, IL-1α, IL-17, TNF-α, MCP-1 and KC/CXCL1, and elevated levels of the protective cytokine IFN-γ when compared with homogenates from B6.Sst1^S^ animals (Fig. 5g and Extended Data Fig. 10f). Furthermore, tSNE analysis revealed an enrichment of AM and IM accompanied by a pronounced reduction in parenchymal neutrophils in B6.Sst1^S^*Bach1*^−/−^ compared with B6.Sst1^S^ mice (Extended Data Fig. 10g).

Together, these studies employing a mouse model that recapitulates the pulmonary tissue necrosis observed in human disease confirmed that Bach1 plays a prominent role in regulating the host antioxidant response to Mtb infection.

### Bach1-deficient B6.Sst1^S^ mice are highly resistant to Mtb

**Fig. 6:**
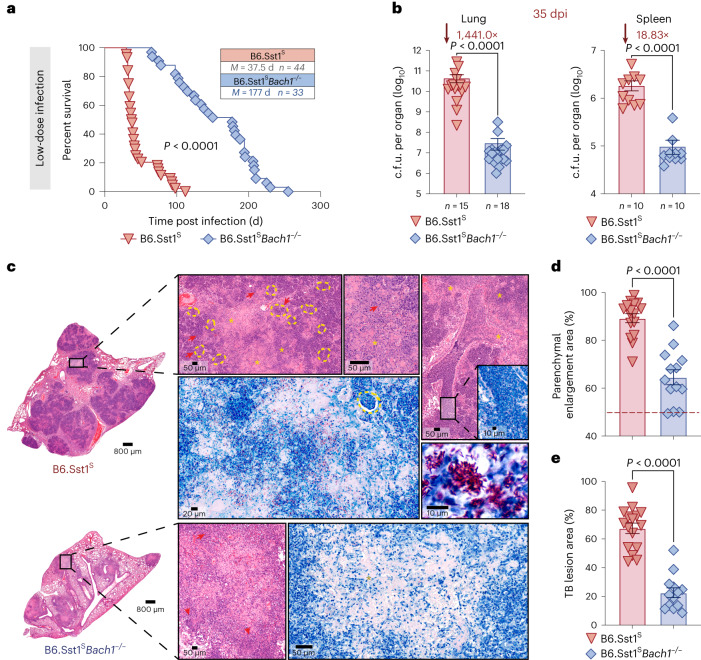
B6.Sst1^S^*Bach1*^−/−^ mice exhibit enhanced host resistance to Mtb infection. **a**–**e**, B6.Sst1^S^ and B6.Sst1^S^*Bach1*^−/−^ mice were infected at low dose with Mtb. **a**, Survival curves of Mtb-infected B6.Sst1^S^ (*n* = 44) and B6.Sst1^S^*Bach1*^−/−^ (*n* = 33) mice. Data pooled from 4 independent experiments (*P* < 0.0001, Mantel–Cox test). **b**, C.f.u.s determined at 35 dpi by plating lung and spleen homogenates onto 7H11 agar plates. Results are pooled from 3 separate experiments (WT *n* = 15 vs *Bach1*^−/−^ *n* = 18 for lungs and WT *n* = 10 vs *Bach1*^−/−^ *n* = 10 for spleens; two-tailed Mann–Whitney *U*-test). **c**, Representative H&E (purple) and ZN (blue) images of lungs from B6.Sst1^S^ (top) and B6.Sst1^S^*Bach1*^−/−^ (bottom) mice. Each image is a composite of sections from 4–6 individual mice per group per experiment (*n* = 13–16 total, 3 experiments performed; two-tailed Mann–Whitney *U*-test). As indicated, B6.Sst1^S^ mice exhibited massive necrotic tissue damage (asterisk) with intrabronchial and intra-alveolar (arrow) accumulation of necrotic cellular material. B6.Sst1^S^*Bach1*^−/−^ mice also showed markedly reduced tissue necrosis (arrow). **d**,**e**, Parenchymal enlargement (**d**) and TB lesion areas (**e**) as measured in the lung sections. Data pooled from 3 independent experiments (B6.Sst1^S^ *n* = 16 vs B6.Sst1^S^*Bach1*^−/−^ *n* = 13; two-tailed Mann–Whitney *U*-test). Data shown in **a**, **b**, **d** and **e** represent mean ±s.e.m. Source data

To further assess the contribution of Bach1 in promoting severe disease, we monitored the outcome of low-dose Mtb infection in the Bach1-expressing or deficient hypernecrotic B6.Sst1^S^ mice studied in the previous section. Bach1 deficiency in B6.Sst1^S^ mice dramatically enhanced host resistance to Mtb infection by extending their median survival time from 37.5 to 177 dpi (Fig. 6a). This increased survival was accompanied by a pronounced >3 log_10_ reduction in bacterial burden in the lungs and 19-fold reduction in spleens of the B6.Sst1^S^*Bach1*^−/−^ animals (Fig. 6b).

In agreement with previous studies^36,37^, extensive cellular necrosis was observed in the lungs of the Mtb-infected B6.Sst1^S^ mice as evidenced by the presence of large numbers of pyknotic nuclei and cellular debris within both bronchial as well as alveolar spaces. The lungs of these animals also displayed substantially increased numbers of AFB per host cell (Fig. 6c top, insets) and in cell-free necrotic areas (Fig. 6c top). In contrast, the B6.Sst1^S^*Bach1*^−/−^ mice exhibited reduced areas with cellular debris in the lung parenchyma as well as fewer AFB (Fig. 6c bottom). Quantitative analysis revealed a major reduction in the size of the lung parenchyma as well as Mtb-infected granulomatous lesions in the B6.Sst1^S^*Bach1*^−/−^ mice compared with B6.Sst1^S^ animals (Fig. 6d,e).

Taken together, the above findings provide additional support for the hypothesis that Bach1 downregulates host resistance to Mtb.

## Discussion

The detrimental effects of the oxidative stress response have been implicated as a major cause of cellular and tissue damage in numerous diseases^11,13,40,41^. Exacerbated lipid peroxidation is one consequence of uncontrolled oxidative stress that has been linked with necrotic cell death through the induction of ferroptosis^39,40^. The transcription factor Nrf2 is a master regulator of host antioxidant response and its activity has been shown to ameliorate a variety of pathological conditions and is expressed in Mtb-infected alveolar macrophages^3^. Here we demonstrate a major role for Bach1, a suppressor of Nrf2 activity, in Mtb infection. Our findings provide additional evidence that uncontrolled lipid peroxidation is a key element in determining TB disease progression through its induction of cellular necrosis and tissue damage.

Previous clinical studies have highlighted the association of oxidative stress with active TB^13,17^. Here while confirming these observations, we uncovered a further correlation of disease severity with *BACH1* expression in blood and lung biopsies. The latter findings were supported by data obtained from parallel analyses of Bach1 levels in both Mtb-infected NHP and mice. Enhanced BACH1 expression has been observed in other human diseases associated with oxidative stress (for example, atherosclerosis, cancer, Down syndrome, Parkinson’s disease)^19,25,26^, suggesting a fundamental role for this transcription factor in pathogenesis. In the case of TB, we observed elevated *BACH1* mRNA expression in infected individuals who later progress to active tuberculosis, suggesting its use as a biomarker for disease risk.

Importantly, when we analysed tissue expression of BACH1 in both Mtb-infected humans and experimentally infected animals, we observed a strong spatial association of the protein with necrotic areas in granulomas. These findings mirror similar observations showing preferential BACH1 expression in areas of tissue damage within human atherosclerotic plaques^25^. The link between Bach1 expression and necrosis in Mtb infection was further evidenced in the effects of Bach1 deficiency on both host resistance and immunopathology in vivo. We observed a dramatic reduction in tissue necrosis evidenced by SytoxGreen DNA staining in vivo accompanied by an enrichment of AM in the lungs of Mtb-infected *Bach1*^−/−^ mice, suggesting that under these conditions, this cell population is resistant to necrosis. Importantly, we found that in Mtb-infected *Bach1*^−/−^ mice, ferroptosis-related genes associated with antioxidant properties were upregulated in different myeloid cell populations (the same cellular subsets displaying elevated Bach1 expression in the lungs of Mtb-infected WT mice), implicating a role for Bach1 in modulating Mtb-induced necrosis in vivo. This hypothesis was supported by our in vitro findings demonstrating that Bach1-deficient BMDMs are partially resistant to Mtb-induced necrosis. Of interest, uninfected Bach1-deficient macrophages in vitro also showed lowered oxidative stress, suggesting that Bach1 is suppressing the host protective antioxidant pathway at baseline. The failure to see a complete ablation of cellular necrosis in vivo and in vitro under conditions of Bach1 deficiency is consistent with a variety of previous studies demonstrating the function of multiple redundant necrotic death modalities in Mtb-infected cells^5–8,10,11,13,42^.

Accompanying its role in cellular necrosis, Bach1 deficiency had a major effect on murine host resistance to Mtb in both BL/6 WT high-dose and B6.Sst1^S^ low-dose infection models. This phenotype was manifested in reduced bacterial burden and lung pathology as well as increased host survival. Our analysis revealed an association of the increased resistance with decreased lipid peroxidation and enhanced Gpx4 and GSH, consistent with the known role of Bach1 in the oxidative response^24^. Further investigation demonstrated major effects of Bach1 deficiency on cytokine/chemokine production-associated tissue inflammation and neutrophil accumulation. Additional analyses revealed a poor representation of a number of gene set enrichment pathways related to extensive tissue inflammation along with the exclusive induction of a pathway related to antimicrobial peptides in the lungs of Mtb-infected B6.Sst1^S^*Bach1*^-/-^ mice compared with Mtb-infected B6.Sst1^S^ animals, suggesting their possible contribution to the increased host resistance. The disease outcome observed here is highly consistent with previous reports on the role of Bach1 in promoting tissue damage and inflammation in murine models of colitis, neurodegenerative diseases, cardiovascular disease, sepsis and lupus erythematosus^27,28,32,43^. In the majority of those studies, the effects of Bach1 deficiency were closely correlated with enhanced antioxidant response and this association was clearly evident in our observation of elevated expression of antioxidant genes related to both GSH and iron metabolism. For example, Bach1-deficient B6.Sst1^S^ mice displayed enhanced expression of *Gclm* which is fundamental for GSH synthesis, as well as *Hmox1* and *Spic* important for iron metabolism by promoting haem degradation and iron efflux, respectively, in macrophages^44,45^. Thus, the regulation of genes related to both GSH and iron metabolism may play an important role in host cell resistance to cellular necrosis upon Mtb infection.

Among the enzymes and co-factors that are known to participate in GSH metabolism, Gpx4 along with its co-factors GSH and selenium plays an important role in preventing necrotic cell death by dampening the accumulation of toxic lipid peroxides on biological membranes^16,46^. We have previously shown that Gpx4 expression is fundamental for host resistance to Mtb infection in vivo and in vitro by preventing the exacerbation of oxidative stress^13^. The findings presented here are consistent with these observations as well as previous reports demonstrating an important role for GSH metabolism in regulating host resistance to Mtb infection^12,47^. While our previous and current work have demonstrated major functions for Gpx4 in host resistance to TB, the enzyme itself is probably a poor target for host-directed therapy of TB, since its depletion or inhibition results in worsened disease outcomes. Since Nrf2 is known as a master upstream regulator of the host antioxidant response including GSH metabolism, the inhibition of its suppressor, Bach1, may provide an alternative strategy for boosting the expression of the enzymes and their co-factors essential for dampening lipid peroxidation-mediated tissue necrosis and enhancing host resistance to TB. Interestingly, Bach1 activity is known to be repressed by its interaction with haem^24^. However, haem by itself is cytotoxic due to its induction of ferroptosis^48,49^, thus ruling out its use as a host-directed therapy for suppressing Bach1 activity. Nevertheless, the discovery and development of other pharmacologically acceptable inhibitors of Bach1 would seem a fruitful strategy, impacting a wide variety of diseases in which lipid peroxidation-mediated cellular necrosis is known as an important mechanism underlying pathogenesis.

## Methods

### Institutional Review Board approval

This work complied with all relevant ethical regulations and we obtained informed consent from all donors. The study protocols were approved by Maternidade Climério de Oliveira Ethics Committee, Federal University of Bahia (protocol number 037/2011, Ethics Committee approval number 034/11). All animal studies were conducted in Assessment and Accreditation of Laboratory Animal Care accredited Biosafety Level 2 and 3 facilities at the NIAID/NIH using a protocol (LPD-99E) approved by the NIAID Animal Care and Use Committee.

### Human study design

*BACH1* expression was tested in 30 healthy control individuals who had TB excluded through clinical and radiological investigation and who were IGRA-negative tested using the QuantiFERON Gold In Tube assay (3rd generation) (Qiagen), and in 30 individuals with culture-confirmed pulmonary TB AFB screening in sputum smears (by microscopy). Supporting sputum cultures (Lowenstein–Jensen solid cultures) were performed in all patients (Supplementary Table 1). Individuals with no symptoms and normal chest X-ray but with positive IGRA tests were clustered in the TBI group. Venous blood was collected in sodium heparin tubes for isolation of PBMC. Cells were cryopreserved at the biorepository of the Laboratory of Inflammation and Biomarkers, Fundação Oswaldo Cruz, Salvador, Brazil. For the immunological assays performed, selected samples from adult (>18-yr-old) HIV-negative individuals with confirmed PTB or controls were matched by age and sex (±5 yr). Sample size was determined on the basis of calculations of study power of 80% (alpha error, 5%) to detect differences in *BACH1* expression >2% (arbitrary cut-off) between TB and healthy controls. Cryopreserved PBMCs were thawed and monocytes were column purified using CD14 beads. RNA extraction was performed using the Qiagen Easy RNA extraction kit. *BACH1* mRNA levels were assessed by real-time PCR and gene fold-increase relative to β-actin (ACTB) was calculated.

*BACH1* primers:

1. Forward sequence: CACAATTCTTCCATAGACCCTC
2. Reverse sequence: TCTGCCACTTCTCGCTCC

*ACTB* primers:

1. Forward sequence: CACCATTGGCAATGAGCGGTTC
2. Reverse sequence: AGGTCTTTGCGGATGTCCACGT

In a separate study, we analysed blood RNA-sequencing data from several timepoints (before diagnosis for progressors) from the Adolescent Cohort Study (ACS, GSE79362), a longitudinal cohort profiling individuals who progressed to active TB (*n* = 37 progressors) or from 106 controls with positive IGRA and/or TST test, who remained TB-free^34^ (Supplementary Table 2). Normalized data from GEO was applied to the multicohort analysis framework to calculate effect sizes reflecting *BACH1* expression differences across clinical groups (controls and TB progressors). Hedge’s *g* was used to measure the effect size.

### Animal experiments

Thy1.1 C57BL/6J, B6.SJL (CD45.1/1) and B6.SJL/C57BL/6 (CD45.1/2) mice (9–12-week-old, male) were obtained through a NIAID supply contract with Taconic Farms (Germantown, New York). Thy1.1 C57BL/6J mice were used as WT C57BL/6J controls. *Bach1*^*−/−*^ mice (C57BL/6J background) were generously provided by Dr Kazuhiko Igarashi (Tohoku University Graduate School of Medicine). B6.Sst1^S^ mice were donated by Dr Igor Kramnik (Boston University, USA) and crossed with *Bach1*^*−/−*^ mice. Animal genotyping was performed by Transnetyx. Animals were housed under specific pathogen-free (SPF) conditions with ad libitum access to food and water, 20–26 °C, 30–70% humidity and a 12 h/12 h light/dark cycle.

Lung samples from Mtb-infected rhesus macaques (ID#: DG3X, DG5F, DGKA, DG9R, DGOI, DF4H, DGRI, DGNK, DG3P and DG4N) initially described in ref. ^35^ were used. Formalin-fixed paraffin-embedded tissue sections from Mtb-infected animals were stained for BACH1.

### Generation of competitive mixed BM chimaeric mice preparation

B6.SJL/C57BL/6 (CD45.1/2) mice were lethally irradiated (900 rad) and reconstituted with a total of 2 × 10^6^ donor BM cells from B6.SJL CD45.1/1 and C57BL/6 CD45.2/2 mice mixed in equal parts. Trimethoprim-sulfamethoxazole water was given to irradiated mice for 4 weeks. Mice were maintained on regular water for 8 additional weeks to ensure complete immune reconstitution before infection.

### Bacterial strains and culture conditions

Mtb H37Rv strain was grown in 7H9 broth (Sigma-Aldrich) supplemented with 0.05% Tween 80 (Thermo Fisher) and 10% oleic acid-albumin-dextrose-catalase (OADC; BD Biosciences) at 37 °C. H37Rv expressing the red fluorescent protein (H37Rv-RFP) was a kind gift from Dr Joel Ernst (University of California San Francisco, USA) and grown in 7H9 broth (BD Biosciences) supplemented with 0.05% Tween 80 (Thermo Fisher), 10% OADC and 30 μg ml^−1^ kanamycin (Sigma-Aldrich) at 37 °C. Bacteria in mid-log phase (optical density (OD) of 0.6–1.0) were centrifuged at 5,000 r.p.m. for 10 min, resuspended in fresh 7H9 media and frozen at −80 °C in aliquots of ~10^8^ bacilli per ml.

### Primary cell cultures

Murine BMDMs were generated by dispersing cells (3–5 × 10^6^ cells) from both femurs and tibiae and seeding them in Petri dishes (100 × 15 mm size) containing 10 ml of BMDM-differentiation media (DMEM/F12 containing 2 mM l-glutamine (Gibco), 10% fetal bovine serum (FBS), 2% HEPES (Life Technologies), 1 mM sodium pyruvate (Gibco), 25 μg ml^−1^ gentamicin (Gibco) and 20% L929-conditioned media) at 37 °C with 5% CO_2_. After 4 d of incubation, 10 ml BMDM-differentiation media without gentamicin was added. On day 6, BMDMs were detached by washing cells with cold 1× PBS.

### In vivo Mtb infection

Animals were infected with H37Rv strain by aerosol at low-dose infection (~100–250 c.f.u. per mouse) or by intrapharyngeal (IPH) inoculation at high dose (~1,000–2,000 c.f.u. per mouse). For the IPH infection, mice were anaesthetized with isoflurane, and in a vertical body position their tongues pulled aside for pharynx exposure. Mtb inoculum (30 μl) was administered intrapharyngeally into the airway. Infection dose was confirmed by plating lung homogenates on Middlebrook 7H11 agar plates supplemented with 0.5% (v/v) glycerol and 10% (v/v) OADC-enrichment media. High-dose Mtb infection was used as a model of Mtb-induced pulmonary necrosis in immunocompetent C57BL/6 mice^11,13^.

### Preparation of single-cell suspension from lungs

Lung lobes isolated from mice were washed with sterile 1× PBS, dissected into small pieces, digested in RPMI medium containing Liberase TL (0.33 mg ml^−1^; Sigma-Aldrich) and DNase I (0.1 mg ml^−1^; Sigma-Aldrich) at 37 °C for 45 min under agitation (200 r.p.m.) and added with FBS to block enzymatic digestion. Lung tissue was dispersed by passage through a 70-μm-pore-size cell strainer. Red blood cells were lysed with ACK buffer (Gibco) at room temperature for 3 min. Lung cells were washed with 1× PBS supplemented with 10% FBS, centrifuged at 1,500 r.p.m. for 5 min and the cell pellet resuspended in RPMI medium supplemented with 10% FBS. Cell numbers were counted using ViaStain acridine orange propidium iodide staining on a Cellometer Auto 2000 cell counter (Nexcelom).

### Flow cytometry

Intravenous injection of 2 μg anti-CD45 (clone 30-F11; Invitrogen) 3 min before euthanasia was performed to distinguish cells within the lung vasculature and parenchyma^13^. Cocktails of conjugated or unconjugated antibodies diluted in 1× PBS containing 2% FBS and 10% Brilliant Stain buffer (BD Biosciences) were added to isolated cells and incubated for 30 min at 4 °C. Antibodies used were directed against CD11b (1:300, clone M1/70), CD11c (1:200, clone HL3), Ly6G (1:100, clone 1A8), CD24 (1:200, clone M1/69), CD19 (1:100, clone 1D3), B220 (1:100, clone RA3-6B2), CD4 (1:100, clone GK1.5 or RM4-5), CD45 (1:100, clone 30-F11), CD45.2 (1:100, clone 104), Siglec-F (1:200, clone E50-2440), TCR-β chain (1:100, clone H57-597) and TCR-γδ (1:100, clone GL3), all purchased from BD Biosciences; F4/80 (1:50, clone BM8) was purchased from Thermo Fisher; CD8-α (1:100, clone 53-6.7), CD11c (1:200, clone N418), CD44 (1:300, clone IM7), CD64 (1:100, clone X54-5/7.1), CD69 (1:100, clone H1.2F3), CD88 (1:100, clone 20/70), IA/IE (1:300, MHCII, clone M5/114), NK1.1 (1:100, clone PK136), CD45.1 (1:100, clone A20) and Ly6C (1:200, clone HK1.4) were purchased from BioLegend; monoclonal rabbit unconjugated Gpx4 (1:100, clone EPNCIR144) was purchased from Abcam. Unconjugated monoclonal rabbit antibody was detected with donkey F(ab’)2 anti-rabbit IgG H&L pre-adsorbed (1:700, Abcam) and rabbit IgG monoclonal antibody (1:100, Abcam) was used as primary isotype control. Ultraviolet Fixable Live/Dead cell stain dye was purchased from Molecular Probes (Invitrogen) and the staining was performed following manufacturer instructions. Samples were acquired on a FACSymphony A5 SORP flow cytometer (BD Biosciences) or an LSR II Fortessa flow cytometer and results were analysed using FlowJo v.10 software (Three Star).

### Determination of glutathione, total antioxidant status and lipid peroxidation levels in lungs

Lungs were homogenized in 1× PBS and centrifuged at 13,000 *g* at 4 °C for 10 min to remove tissue matrix and cell debris. Supernatants were sterilized by 0.22-μm filtration and stored at −80 °C. Glutathione levels, TAS and lipid peroxidation levels were measured using the Glutathione assay kit, the antioxidant assay kit and the TBARS assay kit (all from Cayman), respectively, following manufacturer instructions. Glutathione, TAS and lipid peroxidation levels were normalized on the basis of total protein content determined using Pierce protein assay (Thermo Fisher) according to manufacturer instructions.

### Multiplex cytokine arrays

Levels of cytokines and chemokines in lung tissue homogenates were assessed using a MILLIPLEX MAP mouse cytokine/chemokine magnetic bead panel kit (Millipore Sigma) according to manufacturer instructions and measured using a MAGPIX instrument (R&D Systems). Total protein concentration was measured using Pierce Protein assay (Thermo Fisher) and values were used to normalize cytokine/chemokine levels on the basis of total protein content.

### Histopathology, immunohistochemistry and necrotic tissue detection

For histological analysis, lungs were fixed with 10% formaldehyde, embedded in paraffin, sectioned and stained with hematoxylin and eosin (H&E) or Ziehl-Neelsen (ZN). Samples were examined under light microscopy and images scanned using an Aperio VERSA scanner (Leica Microsystems).

Fixed tissue was paraffin embedded and 10-μm-thick sections were prepared. Slides were deparaffinized and treated with AR6 buffer (Akoya Biosciences) for 20 min at 100 °C. Slides were next placed in AR9 buffer (Akoya Biosciences) at 100 °C and allowed to cool to room temperature for ~45–50 min. Samples were permeabilized using 0.2% TritonX 100 (Millipore Sigma) for 10 min and blocked using fetal calf serum (FCS) and/or isotype-matched non-specific immunoglobulin. Primary antibody against BACH1 (Proteintech) was employed at a dilution of 1:250. Tissues were washed, stained with ImmPRESS HRP horse anti-rabbit staining kit (Vector Laboratories) and counterstained with haematoxylin. Slides were mounted and imaged using an Aperio VERSA scanner (Leica Microsystems). For human lung tissue, epitope retrieval was performed using citrate buffer (pH 6.0) in bath water at 98 °C for 45 min. Following endogenous peroxidase blocking (peroxidase blocking solution, Dako), tissue sections were incubated with BACH1 primary antibody (1:500; Proteintech) for 18 h at 4 °C. After washing with 1× PBS, all sections were then incubated with Advance HRP Link buffer for 20 min and then subjected to an additional round of PBS washing followed by incubation with Advance HRP enzyme (Dako) for 20 min. Chemical reactions were developed with 3,3-diaminobenzidine (Dako) and sections were counterstained with Harris hematoxylin. One representative field per sample (×200) was selected by a single experienced pathologist. All immunomarkers were analysed within these same selected fields.

Necrotic tissue detection in the lungs was assessed by inoculating mice with a solution of SytoxGreen dye (Thermo Fisher) at 50 μM intravenously 10 min before mouse euthanasia. Lungs were then collected, washed with 1× PBS and immediately fixed with 10% formaldehyde at 4 °C for 48 h. SytoxGreen fluorescence in whole lung tissue was examined in a motorized stereo microscope (Leica M205 FA) and images captured with a CFC345 cooled monochrome camera (Leica) using LAS X (Leica Microsystems) software. Images were processed using Imaris 8.4.1 (Bitplane) software, and QuPath v.0.4.0 and Image J v.1.53t for visualization and quantification.

### Bacterial load determination

Mtb burden in the lung and spleen homogenates was assessed by serial dilution and plating onto 7H11 agar Petri dishes supplemented with 0.5% (v/v) glycerol and 10% (v/v) OADC-enrichment media. Bacterial colonies were enumerated after 21 d incubation at 37 °C.

### Lipid peroxidation staining

Lipid peroxidation was measured in lung single-cell suspension as well as in BMDM cultures by using click-iT lipid peroxidation imaging kit (Life Technologies) according to manufacturer instructions. Briefly, cells were incubated with the linoleamide alkyne (LAA) reagent (alkyne-modified linoleic acid) at 37 °C for 1 h and then washed with 1× PBS by centrifugation for 5 min at 450 *g*. Cells were then stained extracellularly with specific antibodies at 4 °C for 30 min to determine their phenotype, followed by incubation with Live/Dead detection reagent as described above. Fixation of these cells was performed by adding cytofix/cytoperm buffer (BD Bioscience) for 1 h at 4 °C, washing and then resuspension in 1× PBS. LAA fluorescence was analysed by flow cytometry.

### RNA-seq, scRNA-seq and data analyses

Single-cell suspensions generated from lungs of Mtb-infected and uninfected mice were used for RNA-seq or scRNA-seq studies. For RNA-seq, total RNA was isolated from the suspensions using the RNeasy Plus mini kit (QIAGEN). For all samples, low-quality bases were removed and adapters were trimmed using Trimmomatic v.0.32. After the quality check, sequences were aligned to the *Mus musculus* genome (GRCm39) using STAR (v.2.7.0). After mapping, the output was converted to count tables with the tximport package in R (4.2.1). The count gene expression matrix was examined using the DESeq2 package in R (4.2.1) to identify DEGs. The changes in gene expression levels were considered significant when statistical test values (false discovery rate (FDR) adjusted *P* value) were lower than 0.05 and the fold change/difference higher than ±1.4. Candidate DEGs were visualized in volcano plots and Venn diagrams using the VennDiagram package in R (4.2.1). The obtained DEGs were scanned with the REACTOME pathway database using the compare Cluster package in R (4.2.1).

For scRNA-seq, equal numbers of live cells from each sample of the *Bach1*^*−/−*^ and C57BL/6 WT groups were pooled, stained with propidium iodide at a dilution of 1:100 and live cells were sorted for each group. From this sorted population, 10,000 cells of each group were loaded per lane on a 10X Genomics Next GEM chip, and single-cell GEMs were generated using a 10× Chromium Controller. Subsequent steps to generate complementary DNA libraries were performed following the 10× Genomics protocol. Libraries were pooled and sequenced on an Illumina NextSeq 2000 system. The sequenced data were processed using Cell Ranger (v.6.1.2) to demultiplex the libraries. The count tables were then further processed and analysed using Seurat (v.4.0) in R (v.4.1.0). Cells were then filtered for less than 8% mitochondrial contamination and data were normalized, scaled and merged. FindVariableFeatures and RunHarmony functions were used to integrate the data. Principal components analysis was performed to find neighbours and clusters, and UMAP reduction was performed with 30 dimensions. FindAllMarkers with a filter of log fold change ≥0.25 and percent of cells expressing the marker ≥0.25 was used to identify gene markers that distinguish the cell clusters, and the clusters were manually assigned cell types on the basis of identified canonical markers (Extended Data Fig. 4). FindMarkers was used to identify DEGs between groups. Genes with a log fold change ≥0.5, percent of cells expressing the marker ≥0.2 and adjusted *P* ≤ 0.05 were considered significant and displayed using a volcano plot. AddModuleScore was used to calculate the average expression of a set of genes in the ferroptosis pathway, and results were visualized using feature and violin plots.

### In vitro macrophage infection and cell death measurement

Mtb was grown in complete 7H9 broth media at 37 °C for 7 d and bacterial concentration determined by spectrophotometry at 600 nm. Mtb cultures were centrifuged at 5,000 r.p.m. for 10 min, resuspended in OptiMEM and sonicated for 30 s to reduce bacterial clumping. BMDMs were exposed to either H37Rv or H37Rv-RFP at an MOI of 10 for 3–4 h, washed three times with 1× PBS and then cultured in fresh OptiMEM media. L929-conditioned media were added to the cultures to a final concentration of 2.5% on days 1 and 3.

Necrotic cell death was evaluated by staining adherent cells with Fixable Viability Dye eFluor780 (eBioscience) according to manufacturer protocol. BMDMs were stained with Live/Dead staining solution (1:500 diluted in 1× PBS) at room temperature for 10 min in the dark and then incubated with anti-CD11b antibody (eBioscience) for an additional 20 min. Macrophages were washed with 1× PBS following centrifugation at 450 *g* for 5 min and fixed with cytofix/cytoperm buffer (eBioscence) for 1 h at 4 °C. Fixed cells were then detached, washed, resuspended in 1× PBS with 1% BSA (MP Biomedicals) and analysed by flow cytometry.

### Mitochondrial superoxide assay

BMDM cultures were washed with Hankʼs balanced salt solution with calcium and magnesium (HBSS/Ca/Mg; Gibco) following centrifugation at 450 *g* for 5 min to remove residual culture media. Cells were stained with 5 μM MitoSOX dye (diluted in HBSS/Ca/Mg; Life Technologies) at 37 °C for 10 min according to manufacturer protocol. Extracellular staining for CD11b as cell fixation was performed as described above. MitoSOX fluorescence intensity was measured using a flow cytometer.

### Quantification and statistical analyses

#### Statistics

The results shown in figure legends are presented as mean ± s.e.m. The sample size (*n*) and numbers of independent experiments for the in vivo experiments are described in the graphics or provided in the figure legends. For in vitro experiments, the number of experimental replicates is described in the figure legend. Cytokine/chemokine levels were normalized to total protein concentration per experiment using GraphPad Prism 9.0 software, and clustered and visualized as a heat map using the R package pheatmap. A publicly available RNA-seq data obtained from https://ogarra.shinyapps.io/tbtranscriptome/ as referred to in ref. ^33^ was re-analysed to determine mRNA levels of *BACH1* in TB patients as well as in different experimental settings in vivo. Statistical analyses were performed with GraphPad Prism 9.0 using either unpaired two-tailed *t*-test for comparison between two groups or one-way analysis of variance (ANOVA) for multiple comparisons. The median values with interquartile ranges were used as measures of central tendency and dispersion, respectively, for parameters whose values exhibited a non-Gaussian distribution. The Mann–Whitney test (for two groups), Kruskal–Wallis test with Dunn’s multiple comparisons or linear-trend post-hoc tests (for more than two groups) were used to compare continuous variables. Statistical differences were considered significant when *P* < 0.05.

#### Figure visualization

Figures were generated in Adobe Illustrator 2023 (v.27.7) and R (4.2.1), incorporating images from BioRender.com.

### Reporting summary

Further information on research design is available in the Nature Portfolio Reporting Summary linked to this article.

### Supplementary information

Supplementary Information Supplementary Tables 1–3.

Reporting Summary

### Source data

Source Data Fig. 1 Statistical source data.

Source Data Fig. 2 Statistical source data.

Source Data Fig. 3 Statistical source data.

Source Data Fig. 4 Statistical source data.

Source Data Fig. 5 Statistical source data.

Source Data Fig. 6 Statistical source data.

Source Data Extended Data Fig. 1 Statistical source data.

Source Data Extended Data Fig. 2 Statistical source data.

Source Data Extended Data Fig. 3 Statistical source data.

Source Data Extended Data Fig. 4 Statistical source data.

Source Data Extended Data Fig. 5 Statistical source data.

Source Data Extended Data Fig. 6 Statistical source data.

Source Data Extended Data Fig. 7 Statistical source data.

Source Data Extended Data Fig. 8 Statistical source data.

Source Data Extended Data Fig. 9 Statistical source data.

Source Data Extended Data Fig. 10 Statistical source data.

## Data Availability

All data supporting the findings of this study are available within the Article, and its Supplementary Information, extended data figures or images have been uploaded in Zenodo (10.5281/zenodo.8357287). Additional data supporting the findings in this study are available from the corresponding authors upon request. All sequence data used in this publication are publicly available through the National Center for Biotechnology Information’s GEO repository under accession number GSE236053 (single-cell RNA-seq) and GSE236853 (RNA-seq). Source data are provided with this paper.

## Code availability

The code used for scRNA-seq analyses in this study is from the Seurat pipeline and is available at https://satijalab.org/seurat/articles/get_started.html. The codes used for RNA-seq analyses in this study are publicly available. Tutorials for performing differential expression analysis are available at https://bioconductor.org/packages/release/bioc/vignettes/DESeq2/inst/doc/DESeq2.html; the enrichment analysis tutorial is available at https://guangchuangyu.github.io/software/clusterProfiler/; heat maps tutorial is available at https://jokergoo.github.io/ComplexHeatmap-reference/book/.
