# Supplementary material for: BACH1 promotes tissue necrosis and Mycobacterium tuberculosis susceptibility
Source: Nat Microbiol. 2023 Dec 8;9(1):120–35. doi: 10.1038/s41564-023-01523-7 (PMC10769877; doi:10.1038/s41564-023-01523-7)
Supplement: Supplementary file 1 — Supplementary Tables 1–3. [file 41564_2023_1523_MOESM1_ESM.pdf]

nature microbiology

Article https://doi.org/10.1038/s41564-023-01523-7

# BACH1 promotes tissue necrosis and *Mycobacterium tuberculosis* susceptibility

In the format provided by the authors and unedited

**Supplementary information for the manuscript**

# BACH1 promotes tissue necrosis and *Mycobacterium tuberculosis* susceptibility

Eduardo P. Amaral[1‡], Sivaranjani Namasivayam[1], Artur T L Queiroz[2], Eduardo Fukutani[2], Kerry L. Hilligan[1], Kate Aberman[1], Logan Fisher[1,3], Caio Cesar B. Bomfim[1], Keith Kauffman[4], Jay Buchanan[4], Leslie Santuo[4], Pedro Henrique Gazzinelli-Guimaraes[5], Diego L. Costa[1,6], Mariane Araujo Teixeira[7], Beatriz Barreto-Duarte[2,7,8], Clarissa Gurgel Rocha[9,10], Monique Freire Santana[11,12,13], Marcelo Cordeiro-Santos[12,13,14], Daniel L. Barber[4], Robert J. Wilkinson[15,16,17], Igor Kramnik[18], Kazuhiko Igarashi[19], Thomas Scriba[20], Katrin D. Mayer-Barber[21], Bruno B. Andrade[2,7,8,9, 22,23,24,25*] and Alan Sher[1*‡].

[1]Immunobiology Section, Laboratory of Parasitic Diseases, NIAID, NIH, Bethesda, MD, USA.
[2]Laboratório de Inflamação e Biomarcadores, Instituto Gonçalo Moniz, Fundação Oswaldo Cruz (FIOCRUZ), Salvador, Bahia, Brazil.
[3]Immunology and Microbial Pathogenesis Program, Weill Cornell Medicine Graduate School of Medical Sciences, New York, NY, USA.
[4]T lymphocyte Biology Section, Laboratory of Parasitic Diseases, National Institutes of Allergy and Infectious Disease, National Institutes of Health, Bethesda, MD, USA.
[5]Helminth Immunology Section, Laboratory of Parasitic Diseases, National Institutes of Allergy and Infectious Disease, National Institutes of Health, Bethesda, MD, USA.
[6]Departmento de Bioquímica e Imunologia, Faculdade de Medicina de Ribeirão Preto, Universidade de São Paulo, Ribeirão Preto, Brazil. Programa de Pós-Graduação em Imunologia Básica e Aplicada, Faculdade de Medicina de Ribeirão Preto, Universidade de São Paulo, Ribeirão Preto, Brazil.
[7]Multinational Organization Network Sponsoring Translational and Epidemiological Research (MONSTER) Initiative, Salvador, Brazil.
[8]Curso de Medicina, Universidade Salvador (UNIFACS), Laureate Universities, Salvador, Brazil.
[9]Department of Pathology, School of Medicine of the Federal University of Bahia, Salvador 40110-909, Bahia, Brazil.
[10]Center for Biotechnology and Cell Therapy, D'Or Institute for Research and Education (IDOR), Sao Rafael Hospital, Salvador 41253-190, Bahia, Brazil.

[11]Departmento de Ensino e Pesquisa, Fundação Centro de Controle de Oncologia do Estado do Amazonas-FCECON, Manaus, AM, Brazil.
[12]Fundação Medicina Tropical Doutor Heitor Vieira Dourado, Manaus, Brazil.
[13]Programa de Pós-Graduação em Medicina Tropical, Universidade do Estado do Amazonas, Manaus, Brazil.
[14]Faculdade de Medicina, Universidade Nilton Lins, Manaus, Brazil.
[15]Wellcome Centre for Infectious Disease Research in Africa, Institute of Infectious Disease and Molecular Medicine, University of Cape Town, Observatory 7925 Town, South Africa.
[16]The Francis Crick Institute, London, London, NW1 1AT, United Kingdom.
[17]Department of Infectious Disease, Imperial College London, W12 0NN United Kingdom.
[18]Boston University School of Medicine, Boston, Massachusetts, USA.
[19]Tohoku University Graduate School of Medicine, Sendai, Japan.
[20]South African Tuberculosis Vaccine Initiative, Institute of Infectious Disease & Molecular Medicine and Division of Immunology, Department of Pathology, University of Cape Town, Observatory, 7925, South Africa.
[21]Inflammation and Innate Immunity Unit, Laboratory of Clinical Immunology and Microbiology, NIAID, NIH, Bethesda, MD, USA.
[22]Curso de Medicina, Escola Bahiana de Medicina e Saúde Pública, Salvador, Bahia, Brazil.
[23]Faculdade de Medicina, Universidade Federal da Bahia, Salvador, Brazil.
[24]Curso de Medicina, Universidade Faculdade de Tecnologia e Ciências (UniFTC), Salvador, Bahia, Brazil.
[25]Division of Infectious Diseases, Department of Medicine, Vanderbilt University School of Medicine, Nashville, TN, United States.
[‡]Corresponding authors: eduardo.amaral@nih.gov ; asher@niaid.nih.gov
*co-senior authorship

**Table 1. Demographic details of Brazilian TB cohort**

| | HC (n=30) | TBI (n=30) | PTB (n=30) | Total (n=90) |
|---|---|---|---|---|
| Participants, n | 30 | 30 | 30 | 90 |
| **Age at baseline, years (Mean, range)** | 32.5 (19-58) | 38.4 (20-67) | 35.3 (19-65) | |
| **Sex** | | | | |
| Female, n (%) | 16 (53.33%) | 15 (50%) | 15 (50%) | 46 |
| Male, n (%) | 14 (46.66%) | 15 (50%) | 15 (50%) | 44 |
| **Ethnicity, n (%)** | | | | |
| Non-white | 25 (83.33%) | 26 (86.66%) | 26 (86.66%) | 77 |
| White | 5 (16.66%) | 4 (13.33%) | 4 (13.33%) | 13 |

**Table 2. Demographic details of TB progressor and control participants. South African cohort**

| | Progressors (n=37) | Control (n=106) | Total (n=143) |
|---|---|---|---|
| ***RNA-Seq Transcriptomic Analysis*** | | | |
| Participants, n | 37 | 104 | 141 |
| **Age at baseline, years (Mean, range)** | 15.5 (12-18) | 15.6 (13-18) | |
| **Sex** | | | |
| Female, n (%) | 26 (70.3%) | 69 (66.3%) | 95 |
| Male, n (%) | 11 (29.7%) | 37 (35.6%) | 48 |
| **Ethnicity, n (%)** | | | |
| Colored (Cape mixed ancestry) | 33 (89.2%) | 97 (93.3%) | 130 |
| Black African | 4 (10.8%) | 9 (8.7%) | 13 |

**Table 3 – List of ferroptosis-related genes (FRG)**

| Ferroptosis-related genes (FRG) | | | |
|---|---|---|---|
| Genes associated with induction of ferroptosis | | Genes associated with inhibition/suppression of ferroptosis | |
| **Gene** | **Reference** | **Gene** | **Reference** |
| Steap3 | 1,2 | Sirt1 | 3,4 |
| Alox5 | 5,6 | Nqo1 | 7,8 |
| Ncoa4 | 9 | Acsl3 | 10 |
| Lpcat3 | 11 | Sqstm1 | 12,13 |
| Aco1 | 14 | Hmox1 | 15 |
| Abcc1 | 16,17 | Gclm | 18,19 |
| Acaca | 20,21 | Gclc | 19,22 |
| Cdo1 | 23 | Aldh3a1 | 24 |
| Nox4 | 25 | Cbs | 26 |
| Zeb1 | 27 | G6pdx | 28 |
| Cryab | 21 | Gss | 19 |
| Dpp4 | 29 | Hspb1 | 30 |
| Acsf2 | 31-33 | Sirt3 | 4,34 |
| Alox12 | 5,35 | Gpx4 | 36 |
| Sat1 | 37 | Nfs1 | 38 |
| Phkg2 | 5 | Mt1 | 39 |
| Bach1 | 15 | Slc40a1 | 13,40 |
| Myb | 13,23 | Fth1 | 13,41,42 |
| Rpl8 | 43,44 | Slc7a11 | 13,33,45 |
| Pebp1 | 46 | Ptgs2 | 13 |
| Alox15 | 5,35 | Aifm2 | 13,47 |
| Chac1 | 2,48,49 | | |
| Cs | 33 | | |
| Acsl4 | 50 | | |
| Fdft1 | 51,52 | | |
| Sqle | 53,54 | | |
| Gls2 | 55 | | |
| Keap1 | 56 | | |
| Cd44 | 57 | | |
| Emc2 | 33,58 | | |

**List of references for FRG:**

1 Yan, Y. *et al.* Downregulated Ferroptosis-Related Gene STEAP3 as a Novel Diagnostic and Prognostic Target for Hepatocellular Carcinoma and Its Roles in Immune Regulation. *Front Cell Dev Biol* **9**, 743046, doi:10.3389/fcell.2021.743046 (2021).

2 Hong, Y., Lin, M., Ou, D., Huang, Z. & Shen, P. A novel ferroptosis-related 12-gene signature predicts clinical prognosis and reveals immune relevancy in clear cell renal cell carcinoma. *BMC Cancer* **21**, 831, doi:10.1186/s12885-021-08559-0 (2021).
3 Su, G., Yang, W., Wang, S., Geng, C. & Guan, X. SIRT1-autophagy axis inhibits excess iron-induced ferroptosis of foam cells and subsequently increases IL-1Beta and IL-18. *Biochem Biophys Res Commun* **561**, 33-39, doi:10.1016/j.bbrc.2021.05.011 (2021).
4 Zeng, J. *et al.* The roles of sirtuins in ferroptosis. *Front Physiol* **14**, 1131201, doi:10.3389/fphys.2023.1131201 (2023).
5 Yang, W. S. *et al.* Peroxidation of polyunsaturated fatty acids by lipoxygenases drives ferroptosis. *Proc Natl Acad Sci U S A* **113**, E4966-4975, doi:10.1073/pnas.1603244113 (2016).
6 Song, S. *et al.* ALOX5-mediated ferroptosis acts as a distinct cell death pathway upon oxidative stress in Huntington's disease. *Genes Dev* **37**, 204-217, doi:10.1101/gad.350211.122 (2023).
7 Bersuker, K. *et al.* The CoQ oxidoreductase FSP1 acts parallel to GPX4 to inhibit ferroptosis. *Nature* **575**, 688-692, doi:10.1038/s41586-019-1705-2 (2019).
8 Wang, T. X. *et al.* Tanshinone functions as a coenzyme that confers gain of function of NQO1 to suppress ferroptosis. *Life Sci Alliance* **6**, doi:10.26508/lsa.202201667 (2023).
9 Santana-Codina, N., Gikandi, A. & Mancias, J. D. The Role of NCOA4-Mediated Ferritinophagy in Ferroptosis. *Adv Exp Med Biol* **1301**, 41-57, doi:10.1007/978-3-030-62026-4_4 (2021).
10 Magtanong, L. *et al.* Exogenous Monounsaturated Fatty Acids Promote a Ferroptosis-Resistant Cell State. *Cell Chem Biol* **26**, 420-432 e429, doi:10.1016/j.chembiol.2018.11.016 (2019).
11 Reed, A. *et al.* LPCAT3 Inhibitors Remodel the Polyunsaturated Phospholipid Content of Human Cells and Protect from Ferroptosis. *ACS Chem Biol* **17**, 1607-1618, doi:10.1021/acschembio.2c00317 (2022).
12 Sun, X. *et al.* Activation of the p62-Keap1-NRF2 pathway protects against ferroptosis in hepatocellular carcinoma cells. *Hepatology* **63**, 173-184, doi:10.1002/hep.28251 (2016).
13 Tang, D., Chen, X., Kang, R. & Kroemer, G. Ferroptosis: molecular mechanisms and health implications. *Cell Res* **31**, 107-125, doi:10.1038/s41422-020-00441-1 (2021).
14 Yao, F. *et al.* Iron regulatory protein 1 promotes ferroptosis by sustaining cellular iron homeostasis in melanoma. *Oncol Lett* **22**, 657, doi:10.3892/ol.2021.12918 (2021).
15 Nishizawa, H. *et al.* Ferroptosis is controlled by the coordinated transcriptional regulation of glutathione and labile iron metabolism by the transcription factor BACH1. *J Biol Chem* **295**, 69-82, doi:10.1074/jbc.RA119.009548 (2020).
16 Cao, J. Y. *et al.* A Genome-wide Haploid Genetic Screen Identifies Regulators of Glutathione Abundance and Ferroptosis Sensitivity. *Cell Rep* **26**, 1544-1556 e1548, doi:10.1016/j.celrep.2019.01.043 (2019).
17 Shi, W. K. *et al.* Construction and validation of a novel Ferroptosis-related gene signature predictive model in rectal Cancer. *BMC Genomics* **23**, 764, doi:10.1186/s12864-022-08996-6 (2022).
18 Wang, S., Wang, H., Zhu, S. & Li, F. Systematical analysis of ferroptosis regulators and identification of GCLM as a tumor promotor and immunological biomarker in bladder cancer. *Front Oncol* **12**, 1040892, doi:10.3389/fonc.2022.1040892 (2022).
19 Stockwell, B. R. *et al.* Ferroptosis: A Regulated Cell Death Nexus Linking Metabolism, Redox Biology, and Disease. *Cell* **171**, 273-285, doi:10.1016/j.cell.2017.09.021 (2017).
20 Song, X. *et al.* PDK4 dictates metabolic resistance to ferroptosis by suppressing pyruvate oxidation and fatty acid synthesis. *Cell Rep* **34**, 108767, doi:10.1016/j.celrep.2021.108767 (2021).
21 Liang, Y. *et al.* A novel survival model based on a Ferroptosis-related gene signature for predicting overall survival in bladder cancer. *BMC Cancer* **21**, 943, doi:10.1186/s12885-021-08687-7 (2021).
22 Kang, Y. P. *et al.* Non-canonical Glutamate-Cysteine Ligase Activity Protects against Ferroptosis. *Cell Metab* **33**, 174-189 e177, doi:10.1016/j.cmet.2020.12.007 (2021).
23 Hao, S. *et al.* Cysteine Dioxygenase 1 Mediates Erastin-Induced Ferroptosis in Human Gastric Cancer Cells. *Neoplasia* **19**, 1022-1032, doi:10.1016/j.neo.2017.10.005 (2017).
24 Okazaki, S. *et al.* Synthetic lethality of the ALDH3A1 inhibitor dyclonine and xCT inhibitors in glutathione deficiency-resistant cancer cells. *Oncotarget* **9**, 33832-33843, doi:10.18632/oncotarget.26112 (2018).
25 Park, M. W. *et al.* NOX4 promotes ferroptosis of astrocytes by oxidative stress-induced lipid peroxidation via the impairment of mitochondrial metabolism in Alzheimer's diseases. *Redox Biol* **41**, 101947, doi:10.1016/j.redox.2021.101947 (2021).
26 Wang, L. *et al.* A pharmacological probe identifies cystathionine beta-synthase as a new negative regulator for ferroptosis. *Cell Death Dis* **9**, 1005, doi:10.1038/s41419-018-1063-2 (2018).
27 Lee, J., You, J. H., Kim, M. S. & Roh, J. L. Epigenetic reprogramming of epithelial-mesenchymal transition promotes ferroptosis of head and neck cancer. *Redox Biol* **37**, 101697, doi:10.1016/j.redox.2020.101697 (2020).
28 Cao, F., Luo, A. & Yang, C. G6PD inhibits ferroptosis in hepatocellular carcinoma by targeting cytochrome P450 oxidoreductase. *Cell Signal* **87**, 110098, doi:10.1016/j.cellsig.2021.110098 (2021).
29 Xie, Y. *et al.* The Tumor Suppressor p53 Limits Ferroptosis by Blocking DPP4 Activity. *Cell Rep* **20**, 1692-1704, doi:10.1016/j.celrep.2017.07.055 (2017).
30 Sun, X. *et al.* HSPB1 as a novel regulator of ferroptotic cancer cell death. *Oncogene* **34**, 5617-5625, doi:10.1038/onc.2015.32 (2015).

31 Luo, L., Zhang, S., Guo, N., Li, H. & He, S. ACSF2-mediated ferroptosis is involved in ulcerative colitis. *Life Sci* **313**, 121272, doi:10.1016/j.lfs.2022.121272 (2023).
32 Xie, Y. *et al.* Ferroptosis: process and function. *Cell Death Differ* **23**, 369-379, doi:10.1038/cdd.2015.158 (2016).
33 Dixon, S. J. *et al.* Ferroptosis: an iron-dependent form of nonapoptotic cell death. *Cell* **149**, 1060-1072, doi:10.1016/j.cell.2012.03.042 (2012).
34 Huang, P. *et al.* SIRT3-mediated autophagy contributes to ferroptosis-induced anticancer by inducing the formation of BECN1-SLC7A11 complex. *Biochem Pharmacol* **213**, 115592, doi:10.1016/j.bcp.2023.115592 (2023).
35 Kim, R. *et al.* Ferroptosis of tumour neutrophils causes immune suppression in cancer. *Nature* **612**, 338-346, doi:10.1038/s41586-022-05443-0 (2022).
36 Ingold, I. *et al.* Selenium Utilization by GPX4 Is Required to Prevent Hydroperoxide-Induced Ferroptosis. *Cell* **172**, 409-422 e421, doi:10.1016/j.cell.2017.11.048 (2018).
37 Ou, Y., Wang, S. J., Li, D., Chu, B. & Gu, W. Activation of SAT1 engages polyamine metabolism with p53-mediated ferroptotic responses. *Proc Natl Acad Sci U S A* **113**, E6806-E6812, doi:10.1073/pnas.1607152113 (2016).
38 Alvarez, S. W. *et al.* NFS1 undergoes positive selection in lung tumours and protects cells from ferroptosis. *Nature* **551**, 639-643, doi:10.1038/nature24637 (2017).
39 Zhang, W., Luo, M., Xiong, B. & Liu, X. Upregulation of Metallothionein 1 G (MT1G) Negatively Regulates Ferroptosis in Clear Cell Renal Cell Carcinoma by Reducing Glutathione Consumption. *J Oncol* **2022**, 4000617, doi:10.1155/2022/4000617 (2022).
40 Ma, S., Henson, E. S., Chen, Y. & Gibson, S. B. Ferroptosis is induced following siramesine and lapatinib treatment of breast cancer cells. *Cell Death Dis* **7**, e2307, doi:10.1038/cddis.2016.208 (2016).
41 Hou, W. *et al.* Autophagy promotes ferroptosis by degradation of ferritin. *Autophagy* **12**, 1425-1428, doi:10.1080/15548627.2016.1187366 (2016).
42 Tian, Y. *et al.* FTH1 Inhibits Ferroptosis Through Ferritinophagy in the 6-OHDA Model of Parkinson's Disease. *Neurotherapeutics* **17**, 1796-1812, doi:10.1007/s13311-020-00929-z (2020).
43 Li, Q. *et al.* Inhibition of neuronal ferroptosis protects hemorrhagic brain. *JCI Insight* **2**, e90777, doi:10.1172/jci.insight.90777 (2017).
44 Zhang, D. *et al.* Evidence of pyroptosis and ferroptosis extensively involved in autoimmune diseases at the single-cell transcriptome level. *J Transl Med* **20**, 363, doi:10.1186/s12967-022-03566-6 (2022).
45 Lei, G. *et al.* The role of ferroptosis in ionizing radiation-induced cell death and tumor suppression. *Cell Res* **30**, 146-162, doi:10.1038/s41422-019-0263-3 (2020).
46 Wenzel, S. E. *et al.* PEBP1 Wardens Ferroptosis by Enabling Lipoxygenase Generation of Lipid Death Signals. *Cell* **171**, 628-641 e626, doi:10.1016/j.cell.2017.09.044 (2017).
47 Doll, S. *et al.* FSP1 is a glutathione-independent ferroptosis suppressor. *Nature* **575**, 693-698, doi:10.1038/s41586-019-1707-0 (2019).
48 Chen, M. S. *et al.* CHAC1 degradation of glutathione enhances cystine-starvation-induced necroptosis and ferroptosis in human triple negative breast cancer cells via the GCN2-eIF2alpha-ATF4 pathway. *Oncotarget* **8**, 114588-114602, doi:10.18632/oncotarget.23055 (2017).
49 Dixon, S. J. *et al.* Pharmacological inhibition of cystine-glutamate exchange induces endoplasmic reticulum stress and ferroptosis. *Elife* **3**, e02523, doi:10.7554/eLife.02523 (2014).
50 Doll, S. *et al.* ACSL4 dictates ferroptosis sensitivity by shaping cellular lipid composition. *Nat Chem Biol* **13**, 91-98, doi:10.1038/nchembio.2239 (2017).
51 Shimada, K. *et al.* Global survey of cell death mechanisms reveals metabolic regulation of ferroptosis. *Nat Chem Biol* **12**, 497-503, doi:10.1038/nchembio.2079 (2016).
52 Huang, R. *et al.* Identification of FDFT1 as a potential biomarker associated with ferroptosis in ccRCC. *Cancer Med* **11**, 3993-4004, doi:10.1002/cam4.4716 (2022).
53 Garcia-Bermudez, J. *et al.* Squalene accumulation in cholesterol auxotrophic lymphomas prevents oxidative cell death. *Nature* **567**, 118-122, doi:10.1038/s41586-019-0945-5 (2019).
54 Tang, W., Xu, F., Zhao, M. & Zhang, S. Ferroptosis regulators, especially SQLE, play an important role in prognosis, progression and immune environment of breast cancer. *BMC Cancer* **21**, 1160, doi:10.1186/s12885-021-08892-4 (2021).
55 Suzuki, S. *et al.* GLS2 Is a Tumor Suppressor and a Regulator of Ferroptosis in Hepatocellular Carcinoma. *Cancer Res* **82**, 3209-3222, doi:10.1158/0008-5472.CAN-21-3914 (2022).
56 Koppula, P. *et al.* A targetable CoQ-FSP1 axis drives ferroptosis- and radiation-resistance in KEAP1 inactive lung cancers. *Nat Commun* **13**, 2206, doi:10.1038/s41467-022-29905-1 (2022).
57 Muller, S. *et al.* CD44 regulates epigenetic plasticity by mediating iron endocytosis. *Nat Chem* **12**, 929-938, doi:10.1038/s41557-020-0513-5 (2020).
58 Cao, J. Y. & Dixon, S. J. Mechanisms of ferroptosis. *Cell Mol Life Sci* **73**, 2195-2209, doi:10.1007/s00018-016-2194-1 (2016).
