# Supplementary material for: BACH1 promotes tissue necrosis and Mycobacterium tuberculosis susceptibility
Source: Nat Microbiol. 2023 Dec 8;9(1):120–35. doi: 10.1038/s41564-023-01523-7 (PMC10769877; doi:10.1038/s41564-023-01523-7)
Supplement: Supplementary file 2 — Reporting Summary [file 41564_2023_1523_MOESM2_ESM.pdf]

nature portfolio

Corresponding author(s): Eduardo P. Amaral
Alan Sher

Last updated by author(s): 2023/09/19

Reporting Summary

Nature Portfolio wishes to improve the reproducibility of the work that we publish. This form provides structure for consistency and transparency in reporting. For further information on Nature Portfolio policies, see our Editorial Policies and the Editorial Policy Checklist.

Statistics

For all statistical analyses, confirm that the following items are present in the figure legend, table legend, main text, or Methods section.

n/a	Confirmed	
☐	☒	The exact sample size (n) for each experimental group/condition, given as a discrete number and unit of measurement
☐	☒	A statement on whether measurements were taken from distinct samples or whether the same sample was measured repeatedly
☐	☒	The statistical test(s) used AND whether they are one- or two-sided<br>*Only common tests should be described solely by name; describe more complex techniques in the Methods section.*
☒	☐	A description of all covariates tested
☐	☒	A description of any assumptions or corrections, such as tests of normality and adjustment for multiple comparisons
☐	☒	A full description of the statistical parameters including central tendency (e.g. means) or other basic estimates (e.g. regression coefficient) AND variation (e.g. standard deviation) or associated estimates of uncertainty (e.g. confidence intervals)
☐	☒	For null hypothesis testing, the test statistic (e.g. F, t, r) with confidence intervals, effect sizes, degrees of freedom and P value noted<br>*Give P values as exact values whenever suitable.*
☒	☐	For Bayesian analysis, information on the choice of priors and Markov chain Monte Carlo settings
☒	☐	For hierarchical and complex designs, identification of the appropriate level for tests and full reporting of outcomes
☒	☐	Estimates of effect sizes (e.g. Cohen's d, Pearson's r), indicating how they were calculated

Our web collection on statistics for biologists contains articles on many of the points above.

Software and code

Policy information about availability of computer code

Data collection	BD FACS LSR II Fortessa and BD FACS SSymphony A5 SORP were used to collect flow cytometric data; Cellometer Auto 2000 Cell Counter was used to count viable cells automatically; MAGPIX Instrument (R&D Systems) was used to collect multiplex data; Aperio VERSA (Leica Microsystems, USA), Aperio digital microscope (Leica Microsystems, Wetzlar, Germany) and motorized stereo microscope Leica M205 FA (Leica, Germany) were used to collect microscopical images. Illumina NextSeq 2000 was used to collect RNAseq scRNaseq
Data analysis	Flowjo (version) 10.8.1 was used to analyze flow cytometric data. GraphPad Prism (version 9) and Microsoft Excel (version 16.7) were used to analyze and visualize the data. Microscopy data was processed using Imaris 8.4.1, LAS X software and Aperio Image Scope software, then analyzed using QuPath (version 0.3.2) and Image J (version 1.53t) for visualization and quantification. Photoshop (version 24.7.1) and imageJ were used to equally adjust brightness and contrast across the entire image. R (version 4.1.0; version 4.2.1) was used to analyze and visualize RNAseq and scRNAseq data as well as Cell Ranger (version 6.1.2), Trimmomatic (version 0.32), Seurat (version 4.0) . Illustrator (version 27.7) was used to arrange all graphs in the same figure. Please see Methods section and main text in manuscript for further detail.

For manuscripts utilizing custom algorithms or software that are central to the research but not yet described in published literature, software must be made available to editors and reviewers. We strongly encourage code deposition in a community repository (e.g. GitHub). See the Nature Portfolio guidelines for submitting code & software for further information.

Data

Policy information about availability of data

All manuscripts must include a data availability statement. This statement should provide the following information, where applicable:

- Accession codes, unique identifiers, or web links for publicly available datasets
- A description of any restrictions on data availability
- For clinical datasets or third party data, please ensure that the statement adheres to our policy

All data supporting the findings of this study are available within the article and its supplementary information, extended data figures or images have been in Zenodo (https://doi.org/10.5281/zenodo.8357287). Additional data supporting the findings in the current study are available from the corresponding authors upon request. All sequence data used in this publication are publicly available through the National Center for Biotechnology Information's GEO repository under accession number GSE236053 (Single-cell RNAseq) and GSE236853 (RNA-seq).

Human research participants

Policy information about studies involving human research participants and Sex and Gender in Research.

Reporting on sex and gender	Female: HC (53.33%); TBI (50%); PTB (50%)
Population characteristics	age (mean, range): HC (32.5, 19-28); TBI (380.4, 20-67); PTB (35.3, 19-65)
Recruitment	Healthy control individuals, who had TB excluded through clinical and radiological investigation and who were IGRA negative tested using the QuantiFERON Gold In Tube (3rd generation) (Qiagen). Subjects with culture-confirmed pulmonary TB AFB screening in sputum smears (by microscopy) and supporting sputum cultures (Lowenstein–Jensen solid cultures) were clustered in TBI group. Individuals with no symptoms, normal chest X-ray, but with positive IGRA tests were clustered in the TBI group. This work complied with all relevant ethical regulations, and we obtained informed consent from all donors.
Ethics oversight	The study protocols were approved by Maternidade Climério de Oliveira Ethics Committee, Federal University of Bahia (protocol number: 037/2011, Ethics committee approval number: 034/11).

Note that full information on the approval of the study protocol must also be provided in the manuscript.

Field-specific reporting

Please select the one below that is the best fit for your research. If you are not sure, read the appropriate sections before making your selection.

☒ Life sciences ☐ Behavioural & social sciences ☐ Ecological, evolutionary & environmental sciences

For a reference copy of the document with all sections, see nature.com/documents/nr-reporting-summary-flat.pdf

Life sciences study design

All studies must disclose on these points even when the disclosure is negative.

Sample size	Sample sizes were not statistically predetermined. Mice per group was based on availability of specific genotypes and prior experience as to typical variability. All experiments were performed with sample sizes based on standard protocols in the field (Mayer-Barber et al Nature 2014).
Data exclusions	No data were excluded from the analysis.
Replication	Experiments were reproducible across multiple experiments (at least twice). Figures shown in this study are representative of the replicates or pooled from all experiments performed, as indicated in each figure legend.
Randomization	For experiments related to mice, age- and sex-matched littermate mice were randomized and used for experiments. Animals were grouped based on their genotyping as WT, knockout, or mutant. These groups were described in the figure legend.
Blinding	The Investigators were not blinded to allocation during experiments and outcome assessment, except for microscopy, RNAseq and scRNAseq experiments, for which samples were de-identified prior to data collection and analysis. The data were objectively collected by instruments, e.g. flow cytometry, automatic cell counter etc, thus avoiding biased interpretation.

Reporting for specific materials, systems and methods

We require information from authors about some types of materials, experimental systems and methods used in many studies. Here, indicate whether each material, system or method listed is relevant to your study. If you are not sure if a list item applies to your research, read the appropriate section before selecting a response.

Materials & experimental systems

n/a	Involved in the study
	☒ Antibodies
☒	☐ Eukaryotic cell lines
☒	☐ Palaeontology and archaeology
	☒ Animals and other organisms
	☒ Clinical data
☒	☐ Dual use research of concern

Methods

n/a	Involved in the study
☒	☐ ChIP-seq
	☒ Flow cytometry
☒	☐ MRI-based neuroimaging

Antibodies

Antibodies used

Anti-mouse Ly6C, BV785, (HK1.4) BioLegend Cat# 128041, RRID:AB_2565852
Anti-mouse CD11c, BV650, (N418) BioLegend Cat# 117339, RRID:AB_2562414
Anti-mouse CD11c, BUV737, (HL3) BD Biosciences Cat# 612796, RRID:AB_2870123
Anti-mouse CD11b, BUV737, (M1/70) BD Biosciences Cat# 612800, RRID:AB_2870127
Anti-mouse CD11b, BUV805, (M1/70) BD Biosciences Cat# 741934, RRID:AB_2871246
Anti-mouse Siglec-F, BV605, (E50-2440) BD Biosciences Cat# 740388, RRID:AB_2740118
Anti-mouse Siglec-F, BV650, (E50-2440) BD Biosciences Cat# 740557, RRID:AB_2740258
Anti-mouse F4/80, SB600, (BM8) Thermo Fisher Scientific Cat# 63-4801-82, RRID:AB_2723154
Anti-mouse CD4, BV510, (RM4-5) BioLegend Cat# 100559, RRID:AB_2562608
Anti-mouse CD4, BV786, (RM4-5) BD Biosciences Cat# 563727, RRID:AB_2728707
Anti-mouse CD4, BUV805, (GK1.5) BD Biosciences Cat# 612900, RRID:AB_2827960
Anti-mouse Ly6G, APCCy7, (1A8) BD Biosciences Cat# 560600, RRID:AB_1727561
Anti-mouse I-A/I-E, AF700, (M5/114.15.2) BioLegend Cat# 107622, RRID:AB_493727
Anti-mouse CD24, BUV496, (M1/69) BD Biosciences Cat# 612953, RRID:AB_2870229
Anti-mouse CD24, BUV395, (M1/69) BD Biosciences Cat# 744471, RRID:AB_2742259
Anti-mouse CD45, BV421, (30-F11) BioLegend Cat# 103134, RRID:AB_2562559
Anti-mouse CD45, BUV395, (30-F11) BD Biosciences Cat# 564279, RRID:AB_2651134
Anti-mouse CD45, SB702, (30-F11) Thermo Fisher Scientific Cat# 67-0451-82, RRID:AB_2662424
Anti-mouse CD45.1, PE, (A-20) BioLegend Cat# 110708, RRID:AB_313497
Anti-mouse CD45.1, APCCy7, (A-20) BioLegend Cat# 110716, RRID:AB_313505
Anti-mouse CD45.2, BV421, (104) BD Biosciences Cat# 562895, RRID:AB_2737873
Anti-mouse CD45.2, BUV396, (104) BD Biosciences Cat# 564616, RRID:AB_2738867
Anti-mouse CD64, PECy7, (X54-5/7.1) BioLegend Cat# 139314, RRID:AB_2563904
Anti-mouse CD64, BV421, (X54-5/7.1) BioLegend Cat# 139309, RRID:AB_2562694
Anti-mouse CD19, BV786, (1D3) BD Biosciences Cat# 563333, RRID:AB_2738141
Anti-mouse CD69, AF488, (H1.2F3) BioLegend Cat# 104516, RRID:AB_492845
Anti-mouse CD44, APC, (IM7) BioLegend Cat# 103012, RRID:AB_312963
Anti-mouse NK1.1, BV510, (PK136) BioLegend Cat# 108738, RRID:AB_2562217
Anti-mouse CD88, PerCPCy5.5, (20/70) BioLegend Cat# 135813, RRID:AB_2750209
Anti-mouse CD8a, APC-Cy7, (53-6.7) BioLegend Cat# 100714, RRID:AB_312753
Anti-mouse TCRb, PECF594, (H57-597) BD Biosciences Cat# 562841, RRID:AB_2737831
Anti-mouse Siglec-F, PECF594, (E50-2440) BD Biosciences Cat# 562757, RRID:AB_2687994
Anti-mouse TCRgd, PECF594, (GL3) BD Biosciences Cat# 563532, RRID:AB_2661844
Anti-mouse B220, PECF594, (RA3-6B2) BD Biosciences Cat# 562290, RRID:AB_11151901
Anti-mouse NK1.1, PECF594, (PK136) BD Biosciences Cat# 562864, RRID:AB_2737850
Donkey F(ab')2 Anti-Rabbit IgG H&L preadsorbed, AF647 Abcam Cat# ab181347, RRID:AB_2892986
anti-mouse/human Glutathione Peroxidase 4 antibody, (EPNCIR144) Abcam Cat# ab125066, RRID:AB_10973901
Anti-mouse/human Bach1 antibody, (polyclonal) Proteintech Cat# 14018-1-AP, RRID:AB_2274498
Rabbit IgG, monoclonal [EPR25A] - Isotype Control, Abcam Cat#ab172730, RRID:AB_2687931

Validation

All of the antibodies are commercially available in Biolegend, eBioscence, BD, Thermo, Abcam and Proteintech and have been validated by the vendors. Statements about validation of each antibody related to their reactivity to Mouse, Rat, Human as well as application and/or validation with knockout mice can be found in the manufacturer's websites.

Animals and other research organisms

Policy information about studies involving animals; ARRIVE guidelines recommended for reporting animal research, and Sex and Gender in Research

Laboratory animals

Mice used in this study were maintained in specific-pathogen-free (SPF) conditions with ad libitum access to food and water at the National Institutes of Health and housed at 20-26oC, with relative humidity 30-70, and were maintained on a 12/12-h light/dark cycle. All animal studies were conducted in Assessment and Accreditation of Laboratory Animal Care accredited Biosafety Level 2 and 3 facilities at the NIAID/NIH using a protocol (LPD-99E) approved by the NIAID Animal Care and Use Committee. The following

	mouse strains were used in this study: Thy1.1 C57BL/6J mice, B6.SJL (CD45.1/1), B6.SJL/C57BL/6 (CD45.1/2), Bach1-/- , B6.Sst1s and B6.Sst1sBach1-/- mice. 9-12 week-old male animals were used in this study.
Wild animals	This study did not involve wild animals
Reporting on sex	Male mice were used in this study due to their availability for the experiments.
Field-collected samples	The study did not involved samples collected from the field.
Ethics oversight	All animal studies were conducted in Assessment and Accreditation of Laboratory Animal Care accredited Biosafety Level 2 and 3 facilities at the NIAID/NIH using a protocol (LPD-99E) approved by the NIAID Animal Care and Use Committee.

Note that full information on the approval of the study protocol must also be provided in the manuscript.

Clinical data

Policy information about clinical studies

All manuscripts should comply with the ICMJE guidelines for publication of clinical research and a completed CONSORT checklist must be included with all submissions.

Clinical trial registration	n/a
Study protocol	protocol number: 037/2011, Ethics committee approval number: 034/11
Data collection	A case–control study of HIV unexposed individuals was performed using cryopreserved PBMC samples and corresponding clinical and epidemiological data obtained from participants enrolled in a translational study performed at the Instituto Brasileiro para Investigação da Tuberculose and at the Hospital Especializado Octavio Mangabeira between December 2015 and January 2018. Venous blood was collected in sodium heparin tubes for isolation of PBMC from a subset of participants. Cells were cryopreserved in liquid nitrogen at the biorepository of the Laboratory of Inflammation and Biomarkers, Fundação Oswaldo Cruz, Salvador, Brazil.
Outcomes	For the immunological assays performed, selected samples from adults (age >18 yo) HIV-negative individuals with confirmed PTB or controls were matched by age and sex (±5 years). Sample size was determined based on calculations of study power of 80% (alpha error, 5%) to detect differences in BACH1 expression >2% (arbitrary set up) between TB and healthy controls.

Flow Cytometry

Plots

Confirm that:

☒ The axis labels state the marker and fluorochrome used (e.g. CD4-FITC).

☒ The axis scales are clearly visible. Include numbers along axes only for bottom left plot of group (a 'group' is an analysis of identical markers).

☒ All plots are contour plots with outliers or pseudocolor plots.

☒ A numerical value for number of cells or percentage (with statistics) is provided.

Methodology

Sample preparation	Lung lobes isolated from mice were washed with sterile 1X PBS, dissected into small pieces and then digested in RPMI containing Liberase TL (0.33mg/ml; Sigma-Aldrich, USA) and DNase I (0.1mg/ml; Sigma-Aldrich, USA) at 37oC for 45 min under agitation (200 rpm), followed by adding FBS to block the enzymatic digestion. Lung tissue was dispersed by passage through a 70-[illegible]m pore-size cell strainer. Red blood cells were lysed with ACK buffer (Gibco, USA) at room temperature for 3 min. Lung cells were washed with 1X PBS supplemented with 10% FBS, centrifuged at 1500 rpm for 5 min and the cell pellet resuspended in RPMI supplemented with 10% FBS. Cell numbers were counted using ViaStain acridine orange propidium iodide staining on a Cellometer Auto 2000 Cell Counter (Nexcelom, USA).<br>Cocktails of fluorescently conjugated or unconjugated antibodies diluted in 1X PBS containing 2% FBS and 10% Brilliant Stain Buffer (BD Biosciences, USA) were added to isolated cells and incubated for 30 min at 4oC. Antibodies used were directed against CD11b (clone M1/70), CD11c (clone HL3), Ly6G (clone 1A8), CD24 (clone M1/69), CD19 (clone 1D3), B220 (clone RA3-6B2), CD4 (clone GK1.5 or RM4-5), NK1.1 (clone PK136), CD45 (clone 30-F11), CD45.2 (clone 104), Siglec-F (clone E50-2440), TCR-β chain (clone H57-597) and TCR-γδ (clone GL3), all were purchased from BD Biosciences; F4/80 (clone BM8), CD45 (clone 30-F11) and FoxP3 (clone FJK-16s) were purchased from Thermo Fisher Scientific; CD8-α (clone 53-6.7), CD11c (clone N418), CD44 (clone IM7), CD45 (clone 30-F11), CD64 (clone X54-5/7.1), CD69 (clone H1.2F3), CD88 (clone 20/70), IA/IE (MHCII, clone M5/114), NK1.1 (clone PK136), CD45.1 (clone A20) and Ly6C (clone HK1.4) were purchased from BioLegend; monoclonal rabbit unconjugated Gpx4 (clone EPNCIR144) was purchased from Abcam. Unconjugated monoclonal rabbit antibody was detected with donkey F(ab')2 Anti-Rabbit IgG H&L pre-adsorbed (Abcam, USA) and rabbit IgG monoclonal (Abcam, USA) was used as primary isotype control. Ultraviolet Fixable Live/Dead cell stain dye was purchased from Molecular Probes-Invitrogen and the staining was performed following the manufacturer's instructions.
Instrument	FACSymphony A5 SORP flow cytometer (BD Biosciences, USA) and LSR II Fortessa flow cytometer
Software	BD FACS Diva (version 9.0) and FlowJo (version 10.8.1) were used for collection and analysis, respectively

Cell population abundance	No cells were sorted for mouse studies. For human studies, CD14+ monocytes were column isolated or total monocytes, neutrophils and lymphocytes were pre-sorted from PBMC for RNA extraction.
Gating strategy	Diagrams are provided in Extended Data figure 2. Doublet cells were excluded initially based on FSC-A/FSC-H and then SSC-A/SSC-W. After that exclusion, Live cells were gated based on their lowered staining for the Live/dead probe. Each population was defined based on their positivity or negativity to well-known and specific cell markers.

☒ Tick this box to confirm that a figure exemplifying the gating strategy is provided in the Supplementary Information.
